# Selection of highly stress-tolerant yeast strains relevant for bioethanol fermentation using predictive growth models

**DOI:** 10.3389/fmicb.2026.1783848

**Published:** 2026-03-18

**Authors:** María Alejandra Canseco Grellet, Joaquín Bautista-Gallego, María Francisca Perera, Karina Inés Dantur, Roberto Marcelo Ruiz, Francisco Noé Arroyo-López

**Affiliations:** 1 Laboratorio de Microbiología, Estación Experimental Agroindustrial Obispo Colombres (EEAOC), Las Talitas, Argentina; 2 Facultad de Ciencias, Universidad de Extremadura (UEx), Badajoz, Spain; 3 Instituto de Tecnología Agroindustrial del Noroeste Argentino (ITANOA), Estación Experimental Agroindustrial Obispo Colombres (EEAOC) —Consejo Nacional de Investigaciones Científicas y Técnicas (CONICET), Las Talitas, Argentina; 4 Departamento de Biotecnología de Alimentos, Instituto de la Grasa (CSIC), Campus Universitario Pablo de Olavide, Seville, Spain

**Keywords:** autochthonous yeast, bioethanol, predictive microbiology, *Saccharomyces cerevisiae*, stress tolerance

## Abstract

**Introduction:**

The growing demand for renewable energy together with the environmental impact of fossil fuels, have intensified global interest in sustainable bioethanol production. *Saccharomyces cerevisiae* is the preferred microorganism for industrial fermentation due to its productivity and stress tolerance, but cumulative stress during successive cycles reduces process efficiency. Therefore, selecting stress-tolerant strains capable of adapting to fluctuating conditions is crucial. Predictive microbiology (PM), which applies mathematical models to predict and quantify microbial responses to environmental factors, remains a valuable but still limited approach in yeast-based bioethanol production.

**Methods:**

In this study, diverse PM models were applied to evaluate the effects of temperature, pH, sucrose, and ethanol concentrations on *S. cerevisiae* strains isolated from industrial fermentations in Tucumán, Argentina. Thirteen native isolates were compared with a commercial reference strain (Calsa).

**Results:**

The primary and secondary models used achieved excellent fits in all cases (*R*^2^ > 0.9), effectively describing and anticipating growth responses under stress conditions relevant to industrial fermentations of the evaluated strains, which displayed wide tolerance ranges suggesting potential suitability for cell recycling and high-density fermentation, pending validation under fermentation conditions. Strains T415, Le384, LF84, and SR350 emerged as promising candidates for further validation in industrial bioethanol fermentations to improve the stability and sustainability of industrial bioethanol production, matching or outperforming the control strain.

**Discussion:**

Overall, this study underscores the usefulness of PM tools for characterizing and selecting native yeast strains with enhanced stress tolerance in local bioethanol production, as they allow for anticipation of physiological behavior in growth-based assays that mimic key industrial stresses, reduction of experimental workload, and strengthen strain selection and evaluation criteria.

## 1 Introduction

High oil prices combined with growing concerns about CO_2_ emissions have increased global interest in renewable energy sources (da Silva Fernández et al., 2022). Biofuels offer the potential to foster environmentally sustainable development, reduce dependence on imported resources, and meet rising energy demands while supporting economic growth (Zhang et al., 2023). Bioethanol remains the world’s leading biotechnological product, both in terms of production volume and economic value; currently, its global annual first-generation production exceeds 100 million m^3^ (Favaro et al., 2019). Argentina ranks among the top producers of biofuels and bioethanol worldwide (Canabarro et al., 2023; Zhang et al., 2023).

The bioethanol production process involves obtaining fermentable sugars, converting them into ethanol through yeast fermentation, and subsequently separating and purifying the alcohol (Kumar and Singh, 2016; Wani et al., 2023). The efficiency of bioethanol production depends on several factors, including the type of yeast used, the sugar concentration in the solution, the accessibility of sugars in the substrate and the temperature and pH conditions during fermentation (Azhar et al., 2017; Wani et al., 2023).

The yeast *Saccharomyces cerevisiae* possesses several desirable industrial traits, including rapid growth, efficient anaerobic glucose metabolism with consequent high ethanol productivity, and strong tolerance to several environmental stress factors such as high ethanol concentrations, low pH, and limited oxygen availability. For these reasons, it is the commonly used organism for industrial ethanol production, which constitutes the largest biotechnological application of yeast (Parapouli et al., 2020; da Silva Fernández et al., 2022).

In bioethanol production processes involving cell recycling, the cumulative stress across successive fermentation batches further intensifies its detrimental effects. Despite the challenges, achieving high ethanol concentrations remains a key objective in industrial settings, as it reduces water consumption (for yeast dilution and must preparation) and decreases energy use during the distillation stage, thereby enhancing the overall sustainability of the process. In distilleries, the maximum ethanol concentration is constrained by the inherent tolerance of the yeast strains used, along with elevated temperatures and acidity, which intensify ethanol-induced stress (Basso et al., 2008, 2019). Under these demanding conditions, optimization of the first-generation process requires yeast strains capable of adapting effectively to environmental fluctuations without compromising their fermentative properties (Favaro et al., 2019).

Karyotyping and other molecular techniques have revealed that indigenous yeast populations often replace the original starter strains in fermentation processes (Basso et al., 2019; Canseco Grellet et al., 2022). Over the course of an entire season, during which multiple fermentation cycles take place, adapted strains can evolve and be naturally selected. Therefore, identifying prevalent and persistent strains in industrial environments is an effective strategy for selecting desirable yeasts with multi-stress-tolerant traits (Basso et al., 2019).

Predictive microbiology (PM) is a valuable tool that uses mathematical models to study and forecast the effects of environmental factors on microbial behavior (Arroyo-López et al., 2012; Stavropoulou and Bezirtzoglou, 2019). It is typically categorized into primary and secondary models; primary models focus on microbial responses over time under constant conditions, while secondary models describe how the parameters of primary models change in response to environmental variables (Baranyi et al., 2024). A wide range of models has been successfully applied to characterize the behavior of various pathogenic and spoilage microorganisms in foods (Stavropoulou and Bezirtzoglou, 2019). In the context of winemaking, a great variety of mathematical models have been employed to monitor yeast growth and assess the risk of microbial contamination in grapes and derived products (Petruzzi et al., 2022). Furthermore, kinetic models such as the Monod and Gompertz equations have been used to estimate fermentation parameters. These models have helped predict microbial behavior during the bioconversion of alternative substrates, such as overripe bananas, using *Pseudomonas mendocina* and *S. cerevisiae* for bioethanol production under controlled environmental conditions (Kularathne et al., 2020). Despite its empirical validation, the use of PM in yeast-based bioethanol production remains limited.

Therefore, the aim of the present study was to apply this modeling approach to evaluate the influence of temperature, pH, sucrose levels, and ethanol concentration on the growth of *S. cerevisiae* strains under conditions relevant to industrial fermentation.

## 2 Materials and methods

### 2.1 Yeast strains and culture conditions

Thirteen autochthonous *S. cerevisiae* strains were evaluated in this study. These strains were previously isolated from fermented must or yeast cream collected during industrial batch fermentation processes at nine bioethanol distilleries located in Tucumán, Argentina, and selected through a stress tolerance screening (Table 1). These isolates had been previously characterized based on their morphological traits and genotyped using PCR-fingerprinting techniques (Canseco Grellet et al., 2022). This work is limited to research and characterization purposes, and any potential future commercial application of these strains would require appropriate access permits and benefit-sharing agreements, in compliance with applicable legislation and the Nagoya Protocol.

**TABLE 1:** *Saccharomyces cerevisiae* strains assayed in this work, isolated from bioethanol fermentation processes in distilleries located in Tucumán, Argentina, and selected through a stress tolerance screening by Canseco Grellet et al. (2022).

Yeast strains	Distillery	Isolation sample^a^	Sucrose (%)^b^	Ethanol (%)^b^		pH^b^		Temperature (°C)^b^	
			35	8	12	2	2.5	40	42.5
BV37	Bella Vista	FM	+++	+++	+	+	+++	+++	+
C73	Concepción	YC	+++	+++	+	+	+++	+++	+
LC376	La Corona	FM	+++	+++	-	+	+++	+++	+
Le155	Leales	FM	+++	+++	+	+	+++	+++	+
Le384	Leales	FM	+++	+++	-	+	+++	+++	++
LF256	La Florida	FM	+++	+++	-	+	+++	+++	++
LF84	La Florida	FM	+++	+++	+	+	+++	+++	+
M53	Marapa	YC	+++	+++	-	+	+++	+++	++
M57	Marapa	YC	+++	+++	+	+	+++	+++	++
SB31	Santa Bárbara	YC	+++	+++	+	+	+++	+++	++
SR350	Santa Rosa	FM	+++	+++	-	+	+++	+++	++
SR8	Santa Rosa	YC	+++	+++	-	+	+++	+++	+
T415	La Trinidad	FM	+++	+++	-	++	+++	+++	+++

*^a^*Yeast Cream (YC) and Fermented Must (FM).

*^b^*Stress tolerance was evaluated using a dilution spot assay on Yeast Malt Peptone Glucose (YMPG) plates supplemented with intrinsic stress factors associated with the fermentation process (sucrose, ethanol, and low pH) and one extrinsic factor (high temperature), following Canseco Grellet et al. (2022). Growth of yeast micro-drops was visually assessed in undiluted inoculum and in three serial dilutions (10^–1^, 10^–2^, 10^–3^). The growth score assigned increased with the highest dilution at which growth was still visible: 0 for absence of growth; 1 when growth was observed only in the undiluted inoculum; 2 or 3 when growth was observed at 10^–1^ or 10^–2^, respectively; and 4 when growth was still observed at 10^–3^. According to these scores, strains were classified as non-tolerant (-: score 0), partially tolerant (+: scores 1–2), moderately tolerant (++: scores > 2 to < 4), and tolerant (+++: score 4).

Yeast cultures were grown on Sabouraud Dextrose Agar (SAB; Oxoid Ltd., Cheshire, United Kingdom) at 30°C for 48 h prior to use. All strains were preserved in SAB broth supplemented with 20% (v/v) glycerol and stored at –80°C. The *S. cerevisiae* strain from a commercial bakery source (Calsa, Tucumán, Argentina), commonly used as a starter culture in ethanol fermentation processes in northwestern Argentina, was employed as control strain.

### 2.2 Experimental design

The growth performance of *S. cerevisiae* strains was systematically evaluated to predict their behavior under several physicochemical stress conditions, typical of industrial fermentation processes, including temperature, pH, sucrose and ethanol concentrations.

Inocula were prepared by transferring a single colony from pure cultures of each strain into 5 mL of Yeast-Malt-Peptone-Glucose broth (YM, Difco, Becton and Dickinson Company, Sparks, United States) adjusted to pH 5.5 and incubated at 30°C during 24 h. Only for sucrose tolerance assays, the colonies were inoculated into 5 mL of Yeast Nitrogen Base (YNB, Difco, Becton and Dickinson Co., Sparks, MD) at pH 5.5 and supplemented with 1% sucrose as sole carbon source.

After incubation, 1 mL of the culture was centrifuged at 9,000 × g for 10 min. Pellets were washed with sterile saline solution (0.9 % NaCl), centrifuged and resuspended in the same solution to reach a final concentration of approximately 7 ± 0.2 log_10_ CFU/mL. All experiments were performed by triplicate using sterile 96-well microplates, incubated at 28°C for 5 days under aerobic conditions. Each well was filled with 20 μL of inoculum and 330 μL of the corresponding culture medium (YM for all assays and YNB for the sucrose test), resulting in an initial optical density (OD) of 0.2 (inoculum level of ≈6.0 log_10_ CFU/mL). Uninoculated wells for each experimental series were also included in the microplate to determine, and consequently subtract, the noise signal. Cell growth was monitored every 2 h using a Bioscreen C automated spectrophotometer (Oy Growth Curves Ab, Finland) with a wide-band filter (420–580 nm) and 5 s of pre-shaking before each reading.

The experimental conditions evaluated were as follows:

Temperature levels: 4, 10, 16, 22, 28, 32, 36, 40, 43, and 46°C.

pH levels: 2, 2.5, 3, 4.5, 5.5, 8.5, 11, 11.5, and 12, adjusted with sulfuric acid or sodium hydroxide as required.

Ethanol levels: 0, 2, 4, 6, 8, 10, 11, 12, 13, 14, 15, 16, 18, and 20% (v/v), adjusted with sterile absolute ethanol (99.8 %, Sigma-Aldrich, Merck KGaA, Darmstadt, Germany).

Sucrose levels: 0, 1, 2, 3, 4, 5, 10, 15, 20, 25, 30, 35, 40, 45, 50, 55, 60, and 70%, prepared in YNB medium and sterilized by filtration (0.2 μm) to prevent caramelization.

Growth data obtained under the different stress conditions were analyzed using the mathematical models described below, which allow for the description and prediction of the strains growth kinetics.

All experiments were performed in triplicate under aerobic conditions using microplate cultures; therefore, the derived parameters represent growth-based proxies rather than direct measurements of ethanol production. These indicators are intended for strain screening based on stress tolerance and should be further validated under anaerobic fermentation conditions with cell recycling.

### 2.3 Predictive modeling of *S. cerevisiae* strains

#### 2.3.1 Fitting of temperature and pH variables

Growth curves obtained under different temperature and pH conditions were modeled using the reparameterized Gompertz equation described by Zwietering et al. (1990), which is one of the equations most commonly used during decades in PM for primary modeling. The mathematical expression is:


y=D×exp{-exp[(μ×maxe)/D×(λ-t)+1]}

where y = ln(OD_t_/OD_0_), with OD_0_ representing the initial optical density and OD_t_ the optical density at time t; D = ln(OD_max_/OD_0_), with OD_max_ is the maximum OD reached; μ_max_ denotes the maximum specific growth rate (h^–1^); and λ the lag phase period (h).

Primary model fitting was performed by nonlinear regression minimizing the residual sum of squares, using the Quasi-Newton algorithm implemented in Statistica 7.0 (StatSoft Inc., Tulsa, OK, United States).

Then, as a secondary model to describe the influence of temperature on μ_*max*_, the Cardinal Temperature Model with Inflection (CTMI) developed by Rosso et al. (1993) was applied:


$${\text{μ}}_{\text{max}}\;\text{=}\;\text{0}\;\text{if}\;\text{T}\;\leq \;{\text{T}}_{\text{min}}\;\text{or}\;\text{T}\;\geq \;{\text{T}}_{\text{max}}$$

$${\text{μ}}_{\text{max}}\;\text{=}\;{\text{μ}}_{\text{op}}{}_{\text{t}}(\text{D/E})\;\text{if}\;{\text{T}}_{\text{min}}\;<\;\text{T}\;<\;{\text{T}}_{\text{max}}$$

D=(T-T)max(T-T)min2∧

E=(T-optT)min[(T-optT)min(T-T)opt-(T-optT)max

(T+optT-min2T)]

In this model, T_min_ is the minimum temperature below which growth is not observed, T_max_ is the maximum temperature above which growth ceases, and T_*opt*_ corresponds to the optimal temperature at which μ_max_ reaches its optimal value (μ_opt_).

Similarly, the relationship between μ_max_ and pH was modeled using the Cardinal pH Model (CPM) proposed by Rosso et al. (1995):


$${\text{μ}}_{\text{max}\;}\text{=}\;\text{0}\;\text{if}\;\text{pH}\;<\;{\text{pH}}_{\text{min}}\;\text{or}\;\text{pH}\;>\;{\text{pH}}_{\text{max}}$$

$${\text{μ}}_{\text{max}}\;\text{=}\;{\text{μ}}_{\text{opt}}\;(\text{F/G})\;\text{if}\;{\text{pH}}_{\text{min}}\;<\;\text{pH}\;<\;{\text{pH}}_{\text{max}}$$

$$\text{F}\;\text{=}\;({\text{pH-pH}}_{\text{min}})(\text{pH}\;{\text{-pH}}_{\text{max}})$$

G=(pH-pH)min(pH-pH)max-(pH-pH)opt2∧

where, pH_min_ and pH_max_ denote the lower and upper pH limits for growth, respectively, while pH_opt_ is the value at which μ_max_ is maximized.

Model parameters for CTMI and CPM were estimated via nonlinear regression, and the goodness-of-fit was assessed by the coefficient of determination (*R*^2^), using the Quasi-Newton algorithm implemented in Statistica 7.0 (StatSoft Inc., Tulsa, OK, United States).

#### 2.3.2 Fitting of ethanol and sucrose variables

To assess the effects of sucrose and ethanol stress, the area under the OD versus time curve (AUC) was used as an integrative indicator of microbial proliferation. This variable correlates positively with final biomass and μ_max_, and negatively with lag phase duration (Arroyo-López et al., 2009). The AUC was calculated over the entire 5-day monitoring period from blank-corrected OD data and computed via numerical integration using OriginPro 7.5 (OriginLab Corporation, Northampton, MA, United States), serving as the response variable in subsequent nonlinear modeling.

Then, as a secondary model to evaluate the dual role of sucrose as a nutrient at low concentrations and an inhibitor at high concentrations, a generalized Monod-type model was employed, originally developed by Luong (1987) and later applied by Arroyo-López et al. (2009). The model is defined as:


A=[(U×S)/(Ks+S)]×[1-(S/S)max]

where A is the AUC, S the sucrose concentration (%), U the maximum predicted AUC, Ks the saturation constant, and S_*max*_ the concentration at which growth is fully inhibited. The sucrose concentration that yields maximum growth (S_opt_) was calculated as:


S=optKs×[(1+D/Ks)0.5∧-1]

where, D represents the difference between S_max_ and Ks.

To quantify growth inhibition in presence of ethanol, the fractional area (fa) was calculated as the ratio of the AUC of the ethanol-treated culture (area_test) to that of the control culture grown without ethanol (area_control):


fa=area⁢_⁢test/area⁢_⁢control

Plotting fa against the logarithm of ethanol concentration (log_10_) yielded a sigmoidal curve, which was successfully fitted using the modified Gompertz function for decay (Lambert and Pearson, 2000):


fa=A+C×exp⁢[-exp⁢(B⁢(x-M))]

where A is the lower asymptote (near zero), C the range (≈1), B the slope, and M the inflection point (log_10_ ethanol concentration). Based on the model parameters, the non-inhibitory concentration (NIC) and minimum inhibitory concentration (MIC) were calculated as:


NIC=10(M-1.718/B)∧

MIC=10(M+1/B)∧

All model parameters were estimated through nonlinear regression using Statistica 7.0, and model fit quality was assessed by the coefficient of determination (R^2^). Models were fitted independently to each biological replicate, and reported parameters correspond to mean ± SD.

#### 2.3.3 Yeast survival under ethanol stress conditions

The experiment was conducted following the protocol described by Perricone et al. (2014). Yeast survival was assessed in stationary-phase cultures using plate count analysis after 48 h of incubation at 30°C on YM agar.

Samples evaluated corresponded to yeast cultures previously grown in YM broth supplemented with ethanol at final concentrations of 8, 12, 16, and 20% (v/v) and incubated for 5 days at 28°C, as well as YM broth without ethanol, inoculated and incubated under the same conditions (control). Survival data were expressed as the viability index (V.I.), calculated using the following equation:


V.I.=(LogN10/tLogN10)0×100

where N_t_ was yeast concentration for each time and N_0_ the initial inoculum.

### 2.4 Statistical analysis

To determine statistical differences among *S. cerevisiae* strains, a general linear model was fitted for each variable with a main effect of yeast strains and a blocking effect, a variance function VarIdent, was fitted. The DGC test with α of 0.05 was used for mean comparisons. The dependent variables included in the analysis were the ethanol tolerance parameters (NIC, MIC and VI), sucrose response parameters (S_*max*_ and S_*opt*_), and CTMI and CPM parameters derived from the secondary modeling of growth inhibition by temperature and pH, respectively. Analyses were performed using Infostat and Navure software (Di Rienzo et al., 2002).

## 3 Results

### 3.1 Growth of *S. cerevisiae* strains under temperature stress

A total of 420 growth curves were modeled for yeast strains exposed to a broad range of temperatures (4–46°C), using nonlinear regression based on the reparameterized Gompertz function, employed as an empirical tool for estimating kinetic parameters (Supplementary Table 1). In all evaluated conditions, the μ_*max*_ parameter exhibited a systematic and comparable increase across strains as both temperatures increased until the optimum value was reached. The primary Gompertz model showed an excellent fit, with *R*^2^ values ranging from 0.9433 to 0.9985 and RMSE values between 0.0164 and 0.3880. The CTMI secondary model showed high accuracy, with R^2^ ranging from 0.8225 to 0.9734 and RMSE values between 0.0253 and 0.0690 for temperature-related data. As illustrated in Supplementary Figure 1, the CTMI model fitting for four representative wild (selected considering their differences in behavior) *S. cerevisiae* strains reveals similarities among them, particularly regarding their optimal and maximum growth temperatures, as well as differences in their μ_*max*_ values. All strains exhibited growth at temperatures up to 44°C, with optimal temperatures around 30°C and an average peak growth rate of approximately 0.19 h^–1^, ranging from 0.14 to 0.23 h^–1^. The minimum temperatures required to support detectable growth ranged from 6.34 to 7.58°C (Supplementary Figure 1 and Table 2).

**TABLE 2:** Growth parameters for autochthonous and control *S. cerevisiae* strains as a function of temperature, estimated using the cardinal temperature model with inflection.

Strain	μ _max_ (h^–1^)		*T*_min_ (°C)		*T*_opt_ (°C)		*T*_max_ (°C)	
	Mean ± SD	CI 95%	Mean ± SD	CI 95%	Mean ± SD	CI 95%	Mean ± SD	CI 95%
Calsa	0.21 ± 0.00 B	0.16–0.25	7.46 ± 0.13 A	1.24–13.68	31.86 ± 0.67 A	27.75–35.96	45.28 ± 0.22 A	43.19–47.37
BV37	0.21 ± 0.01 B	0.13–0.29	6.63 ± 0.17 A	0.00–15.22	29.74 ± 0.83 B	23.60–36.11	44.39 ± 0.03 D	41.25–47.50
C73	0.21 ± 0.00 B	0.14–0.29	6.44 ± 0.28 A	0.00–15.71	29.71 ± 0.14 B	23.43–35.99	44.58 ± 0.02 C	41.41–47.76
LC376	0.16 ± 0.01 D	0.09–0.22	6.34 ± 0.44 A	0.00–19.89	30.60 ± 0.67 B	22.89–38.31	45.63 ± 0.22 A	41.17–50.09
Le155	0.20 ± 0.01 B	0.12–0.28	7.58 ± 0.44 A	0.00–15.78	30.59 ± 0.14 B	24.04–37.13	44.78 ± 0.02 B	41.35–48.21
Le384	0.19 ± 0.00 C	0.13–0.24	7.14 ± 0.28 A	1.11–13.17	30.21 ± 0.14 B	25.00–35.43	44.93 ± 0.22 B	42.11–47.75
LF84	0.21 ± 0.00 B	0.12–0.29	7.21 ± 0.28 A	0.00–17.72	30.93 ± 0.14 A	23.66–38.19	44.76 ± 0.02 B	41.22–48.30
LF256	0.21 ± 0.01 B	0.13–0.28	6.49 ± 0.13 A	0.00–16.30	30.05 ± 0.14 B	23.54–36.57	44.94 ± 0.22 B	41.47–48.41
M53	0.18 ± 0.01 C	0.12–0.24	6.93 ± 0.17 A	0.12–13.44	29.63 ± 0.18 B	24.28–34.72	44.38 ± 0.03 D	41.48–47.36
M57	0.14 ± 0.01 E	0.09–0.19	7.40 ± 0.17 A	0.00–15.29	30.42 ± 0.18 B	24.92–36.55	44.36 ± 0.03 D	41.71–47.41
SB31	0.23 ± 0.00 A	0.16–0.29	7.01 ± 0.13 A	0.00–15.80	31.45 ± 0.67 A	26.01–36.88	45.08 ± 0.22 A	42.51–47.65
SR8	0.18 ± 0.00 C	0.12–0.24	7.08 ± 0.28 A	0.71–13.46	29.59 ± 0.14 B	23.75–35.44	44.43 ± 0.02 D	41.35–47.52
SR350	0.20 ± 0.01 B	0.12–0.27	6.96 ± 0.13 A	0.00–15.25	30.10 ± 0.14 B	23.54–36.66	44.67 ± 0.22 C	41.27–48.06
T415	0.18 ± 0.01 C	0.12–0.25	6.57 ± 0.44 A	0.00–14.30	30.08 ± 0.67 B	24.08–36.09	45.13 ± 0.02 A	41.74–48.52

Parameters include the maximum specific growth rate (μ_max_, in h^–1^), minimum growth temperature (T_min_), optimum temperature (T_opt_), and maximum temperature (T_max_) for growth (all in °C). Values represent the mean ± standard deviation from three independent replicates fitted independently. Letters indicate statistically significant groupings determined by the DGC test (*p* < 0.05); strains sharing the same letter within a column do not differ significantly from each other.

The kinetic growth evaluation of thirteen autochthonous *S. cerevisiae* strains revealed relevant variations in growth parameters when compared to the industrial control strain Calsa under variable temperature conditions. Strain SB31 exhibited a μ_*max*_ (0.23 h^–1^) significantly higher than the value detected for Calsa (0.21 h^–1^), indicating a greater replication potential under optimal conditions. In contrast, strains such as M57 (0.14 h^–1^), LC376 (0.16 h^–1^), and M53 (0.18 h^–1^) showed significantly lower values. Other strains, including BV37, C73, LF84, LF256, Le155, and SR350, displayed growth rates similar to Calsa, with no significant differences (Table 2).

Regarding the T_*min*_, no significant differences were observed between Calsa (7.46°C) and the autochthonous strains. The values ranged from 6.34°C in LC376 to 7.58°C in Le155, suggesting that all strains have comparable cold tolerance, initiating growth within similar temperature ranges (Table 2).

Respect to the T_*opt*_, SB31 (31.45°C) and LF84 (30.93°C) showed similar performance to Calsa (31.86°C) without significant differences. However, most of the remaining strains, such as BV37, C73, M53, SR8, and LF256, evidenced significantly lower T_*opt*_, ranging between 29.59 and 30.6°C.

In terms of the T_*max*_, LC376 (45.63°C), T415 (45.13°C), and SB31 (45.08°C) reached values similar to Calsa (45.28°C) with no significant differences. In contrast, strains such as BV37 (44.39°C), M53 (44.38°C), M57 (44.36°C), and SR8 (44.43°C) exhibited significantly lower thermal limits.

Based on the applied model, LC376, T415, and SB31 strains exhibit equal or superior responses to the Calsa strain in specific parameters related to thermal stress (Table 2).

### 3.2 Growth of *S. cerevisiae* strains under pH stress

For the pH assay, yeast growth curves were analyzed using the same primary modeling approach employed for the temperature test (reparameterized Gompertz function) (Supplementary Table 2). The primary Gompertz model showed a strong fit, with *R*^2^ values ranging from 0.9153 to 0.9988 and RMSE values between 0.0112 and 0.1486. The variation in μ_*max*_ in response to pH changes was effectively represented using the CPM secondary modeling approaches, with *R*^2^ ranging from 0.8589 to 0.9840 and RMSE values between 0.0139 and 0.0530 for pH-related data. Strains were capable of growing across a broad pH spectrum, from as low as 1.93 to as high as 12.93. Within this range, μ_*max*_ values remained close to 0.21 h^–1^, with observed values ranging between 0.13 and 0.26 h^–1^ (Supplementary Figure 2 and Table 3).

**TABLE 3:** Growth parameters for autochthonous and control *S. cerevisiae* strains as a function of pH, estimated using the cardinal pH model.

Strain	μ _max_ (h^–1^)		*pH*_min_		*pH*_opt_		*pH*_max_	
	Mean ± SD	CI 95%	Mean ± SD	CI 95%	Mean ± SD	CI 95%	Mean ± SD	CI 95%
Calsa	0.19 ± 0.02 C	0.14–0.25	2.13 ± 0.02 A	1.62–2.64	6.01 ± 0.40 A	4.49–7.53	11.57 ± 0.04 C	10.76–12.38
BV37	0.23 ± 0.01 B	0.17–0.29	2.12 ± 0.01 A	1.66–2.59	6.06 ± 0.14 A	4.67–7.45	11.58 ± 0.01 C	10.86–12.30
C73	0.22 ± 0.02 B	0.15–0.30	2.08 ± 0.04 B	1.33–2.78	6.54 ± 0.52 A	4.68–8.62	12.93 ± 0.89 A	10.71–14.32
LC376	0.23 ± 0.01 B	0.18–0.29	2.15 ± 0.01 A	1.73–2.56	6.21 ± 0.17 A	5.00–7.42	11.62 ± 0.06 C	11.01–12.24
Le155	0.24 ± 0.02 B	0.19–0.29	2.13 ± 0.00 A	1.76–2.49	6.10 ± 0.30 A	4.98–7.22	11.68 ± 0.10 C	11.07–12.30
Le384	0.18 ± 0.01 C	0.14–0.22	2.06 ± 0.02 B	1.76–2.35	5.08 ± 0.25 B	3.93–6.23	11.53 ± 0.06 C	10.64–12.43
LF84	0.23 ± 0.01 B	0.17–0.28	2.03 ± 0.05 B	1.46–2.61	6.67 ± 0.29 A	5.26–8.07	12.21 ± 0.05 B	11.30–13.13
LF256	0.18 ± 0.00 C	0.12–0.23	2.11 ± 0.01 A	1.69–2.53	5.22 ± 0.09 B	3.48–6.96	11.73 ± 0.29 C	10.26–13.21
M53	0.22 ± 0.01 B	0.18–0.26	2.07 ± 0.03 B	1.68–2.47	6.21 ± 0.49 A	5.16–7.27	11.72 ± 0.02 C	11.16–12.28
M57	0.13 ± 0.01 D	0.10–0.17	2.01 ± 0.05 B	1.47–2.56	6.20 ± 0.53 A	4.76–7.63	11.84 ± 0.02 C	11.02–12.67
SB31	0.22 ± 0.01 B	0.18–0.26	2.13 ± 0.01 A	1.80–2.46	6.25 ± 0.04 A	5.20–7.30	12.17 ± 0.11 B	11.41–12.93
SR8	0.26 ± 0.00 A	0.20–0.32	2.06 ± 0.04 B	1.52–2.60	6.49 ± 0.23 A	5.16–7.82	11.78 ± 0.11 C	11.13–12.43
SR350	0.24 ± 0.01 B	0.15–0.32	1.93 ± 0.04 C	0.94–2.93	6.46 ± 0.09 A	4.37–8.54	11.62 ± 0.00 C	10.74–12.50
T415	0.20 ± 0.01 C	0.17–0.24	2.06 ± 0.05 B	1.65–2.46	6.21 ± 0.22 A	5.13–7.29	11.79 ± 0.08 C	11.21–12.37

Parameters include the maximum specific growth rate (μ_max_, in h^–1^), minimum growth pH (pH_min_), optimum pH (pH_opt_), and maximum pH (pH_max_) for growth. Values represent the mean ± standard deviation from three independent replicates fitted independently. Letters indicate statistically significant groupings determined by the DGC test (*p* < 0.05); strains sharing the same letter within a column do not differ significantly from each other.

The evaluation of the autochthonous *S. cerevisiae* strains under varying pH conditions also revealed significant differences in tolerance ranges compared to the control strain Calsa. With respect to μ_*max*_, Calsa showed a value of 0.19 h^–1^, comparable to strains such as Le384, LF256 and T415. The most of the strains such as BV37 (0.23 h^–1^), C73 (0.22 h^–1^), LC376 (0.23 h^–1^), Le155 (0.24 h^–1^), LF84 (0.23 h^–1^), M53 (0.22 h^–1^), SB31 (0.22 h^–1^), and SR350 (0.24 h^–1^) showed significantly faster growth rates. In contrast, M57 (0.13 h^–1^) strain showed significantly lower growth rates, suggesting a reduced capacity to proliferate in environments with varying pH levels (Table 3).

Regarding the growth at pH_*min*_ value, Calsa exhibited a value of 2.13, significantly higher than several strains such as C73 (2.05), Le384 (2.06), LF84 (2.03), M53 (2.07), M57 (2.01), SR8 (2.06), SR350 (1.93), and T415 (2.06). Strains such as BV37, LC376, Le155, LF256, and SB31 evidenced similar values than Calsa. In terms of the growth at pH_*opt*_, Le384 (5.08) and LF256 (5.22) strains presented significantly lower values than Calsa (6.01) (Table 3).

When analyzing the growth at pH_*max*_, several autochthonous strains significantly exceeded the value observed in Calsa (11.57), C73 (12.51), LF84 (12.21), and SB31 (12.17) (Table 3).

Yeast strains M57, LF84, C73, Le384, SR8, T415, and M53 showed greater tolerance to low pH conditions, with the SR350 strain being the most tolerant (Table 3).

### 3.3 Growth of *S. cerevisiae* strains under sucrose stress

The growth behavior of *S. cerevisiae* strains was evaluated across a sucrose concentration gradient (0–70%) using the Luong model (1987), given that sucrose acts as a carbon source at low concentrations and as a metabolic inhibitor at higher levels. At low sucrose levels, an increase in AUC was observed with rising concentrations, reaching a defined maximum before declining progressively with further increases in sucrose (Supplementary Figure 3). In most strains, higher AUC values were recorded at approximately 5% sucrose, while concentrations above 70% resulted in complete growth inhibition. Strain M57 deviated markedly from this pattern, exhibiting the lowest AUC among all strains, with a maximum at 2.99% sucrose and total growth inhibition occurring above 62.24%.

The model fitted using a non-linear regression procedure showed a high correlation with the experimental data, where the proportion of variance explained (R^2^) ranging from 0.9389 to 0.9886 and RMSE values between 9.4686 and 22.8817. Thus, the model accurately represents the observed behavior, allowing a precise description of the relationship between yeast growth and sucrose concentration. Consequently, obtained parameters (U, S_*opt*_, S_*max*_, and Ks) (Table 4) can be used for a quantitative comparison of the strains’ responses to different substrate concentrations.

**TABLE 4:** Parameter values estimated using the Luong (1987) model for the *S. cerevisiae* strains grown under sucrose-gradient assays in this study.

Strain	*U*		*Ks* (%w/v)		*S_*max*_ (%w/v)*		*S_*opt*_ (%w/v)*	
	Mean ± SD	CI 95%	Mean ± SD	CI 95%	Mean ± SD	CI 95%	Mean ± SD	CI 95%
Calsa	227.0 ± 3.6 A	193–256	0.80 ± 0.02 A	0.36–1.22	71.55 ± 0.59 C	65.16–77.95	6.81 ± 0.09 A	6.71–6.91
BV37	228.1 ± 0.7 A	212–244	0.51 ± 0.02 C	0.29–0.73	70.09 ± 0.36 D	65.84–74.34	5.49 ± 0.09 C	5.36–5.65
C73	230.8 ± 0.7 A	214–248	0.48 ± 0.02 C	0.28–0.68	71.25 ± 0.19 C	66.88–75.63	5.36 ± 0.09 C	5.22–5.49
LC376	226.0 ± 0.7 A	206–243	0.51 ± 0.02 C	0.28–0.76	70.39 ± 0.19 D	65.52–75.27	5.50 ± 0.09 C	5.35–5.59
Le155	228.4 ± 0.7 A	208–248	0.53 ± 0.02 C	0.26–0.82	72.88 ± 0.36 A	67.64–78.13	5.71 ± 0.09 C	5.49–5.98
Le384	219.8 ± 0.7 C	205–237	0.50 ± 0.02 C	0.30–0.72	70.94 ± 0.19 C	66.63–75.25	5.47 ± 0.09 C	5.27–5.63
LF84	227.2 ± 1.6 A	209–248	0.62 ± 0.02 B	0.39–0.92	71.20 ± 0.19 C	66.39–76.02	6.05 ± 0.09 B	5.93–6.20
LF256	209.4 ± 1.6 D	193–226	0.34 ± 0.02 E	0.17–0.53	73.61 ± 0.36 A	68.80–78.42	4.67 ± 0.09 E	4.55–4.89
M53	229.9 ± 1.6 A	213–250	0.53 ± 0.02 C	0.30–0.77	71.94 ± 0.19 B	67.03–76.85	5.65 ± 0.09 C	5.58–5.70
M57	98.7 ± 1.6 E	84–119	0.16 ± 0.02 F	0.00–0.50	62.24 ± 0.36 G	55.58–68.89	2.99 ± 0.23 F	2.59–3.52
SB31	201.0 ± 4.4 D	191–217	0.29 ± 0.02 E	0.11–0.48	71.97 ± 0.24 B	64.76–76.85	4.26 ± 0.29 E	3.97–4.53
SR8	217.0 ± 1.6 C	199–235	0.43 ± 0.02 D	0.23–0.63	68.04 ± 0.19 F	63.89–72.19	4.97 ± 0.11 D	4.91–5.05
SR350	225.4 ± 1.6 A	208–244	0.52 ± 0.02 C	0.29–0.76	70.20 ± 0.19 D	65.70–74.71	5.54 ± 0.09 C	5.46–5.68
T415	223.2 ± 0.7 B	205–242	0.48 ± 0.02 C	0.25–0.74	69.22 ± 0.19 E	64.33–74.11	5.30 ± 0.09 C	5.22–5.43

U refers to the maximum area under the growth curve as estimated by the model; Ks denotes the Monod constant, representing the substrate concentration at which the growth rate is half of its maximum; S_max_ is the highest substrate concentration that fully inhibits microbial growth; S_opt_ is the substrate concentration at which the maximum area value (U) is achieved. Values represent the means of three replicates ± standard deviation fitted independently. Letters indicate statistically significant groupings determined by the DGC test (*p* < 0.05); strains sharing the same letter within a column do not differ significantly from each other.

Regarding the U value, strains such as C73 (230.82), M53 (229.94), Le155 (228.4), BV37 (228.09), and LF84 (227.21) reached highest values. In contrast, strains such as LF256 (209.39), SB31 (200.97), and especially M57 (98.73) showed significantly lower values, reflecting limited growth efficiency under the evaluated conditions. The control strain Calsa exhibited an intermediate U value (227), statistically comparable to the most efficient strains.

Respect to the Ks, strains M57 (0.16), LF256 (0.34), SB31 (0.29), and SR8 (0.43) showed the lowest values, suggesting a higher substrate affinity and better adaptation to substrate-limited conditions. Other strains such as LF84 (0.62) and Le155 (0.53) evidenced intermediate values. In contrast, Calsa displayed a significantly higher Ks value (0.8).

In terms of the S_*max*_, LF256 (73.61%) and Le155 (72.88%) strains showed the highest values, indicating strong tolerance to inhibitory conditions. M57 (62.24%) and SR8 (68.04%) were among the most sensitive. Under these conditions, Calsa demonstrated a S_*max*_ value of 71.55%, below the most tolerant strains but above the least resistant ones.

Finally, for S_*opt*_, LF84 (6.05) and M53 (5.65) strains showed the highest values, while strains such as LF256 (4.67) and M57 (2.99) reached their maximum growth at lower substrate concentrations. As for the control strain Calsa, it presented the highest S_*opt*_ value (6.81).

Le155 and LF256 strains presented better tolerance and higher growth efficiency under inhibitory conditions, in contrast to strain M57, which showed significantly lower growth and greater sensitivity to high sucrose concentrations.

### 3.4 Growth inhibition profiles and ethanol tolerance parameters of *S. cerevisiae* strains

The growth of yeast strains was evaluated under ethanol-induced stress conditions (0–20% v/v) and compared to growth in ethanol-free control medium. The resulting fa versus ethanol concentration (log_10_) plots consistently exhibited a characteristic sigmoidal decay curve. This pattern indicates that yeast susceptibility to ethanol follows a nonlinear dose-response relationship. The entire sigmoidal curve can be clearly divided into three distinct regions: (i) concentrations ranging from zero up to the NIC, where no inhibitory effect was observed and fa remained close to 1; (ii) concentrations between NIC and MIC, where growth inhibition occurred progressively and fa decreased accordingly; and (iii) concentrations above the MIC, where no growth was detected relative to the control and fa approached 0. These regions are denoted in Supplementary Figure 4 as the No Inhibition Region (NIR), Partial Inhibition Region (PIR), and No Growth Region (NGR), respectively for the four representative strains. The response of these strains to increasing ethanol concentrations can be clearly inferred from the fa vs. ethanol curves, as follows: (1) greater ethanol sensitivity is indicated by curves that begin to decline at lower ethanol concentrations (*x*-axis), as observed for strain M57; (2) a broader range of progressive inhibition (NIC–MIC), reflected by a shallower slope, also evident in M57; and (3) higher ethanol tolerance, demonstrated by a curve slightly shifted to the right, as seen for strain Le384. Curve fitting was reliable across all 42 experimental curves analyzed (14 strains in triplicate), yielding *R*^2^ values ranging from 0.9641 to 0.9980, with RMSE values between 0.0296 and 0.1206.

The analysis of NIC and MIC values obtained for the *S. cerevisiae* strains revealed distinct patterns of ethanol tolerance. Significant differences were observed in NIC values, indicating variable initial sensitivity to ethanol among the strains. In contrast, MIC values were more homogeneous, suggesting that maximum ethanol resistance was more similar across most strains (Table 5). The strains exhibiting the lowest sensitivity to ethanol (i.e., the highest NIC values) were BV37, Le384, LF84, SR350, and T415, followed by SR8 and Le155. In contrast, M57 strain evidenced the lowest NIC value (6.32 ± 0.19% v/v). Strain LC376 also showed a relatively low NIC (7.61 ± 0.24% v/v), and both strains differed significantly from the others. In terms of ethanol resistance, as determined by MIC values, strains LC376, Le384, and M53 exhibited the highest tolerance, with MIC values of 14.84 ± 0.23, 14.85 ± 0.09, and 14.97 ± 0.09% v/v, respectively. These values were significantly higher than those obtained for the other strains, demonstrating that these three were the most resistant to ethanol-induced growth inhibition. In contrast, the Calsa control strain was classified among the five least ethanol-tolerant strains, none of which showed statistically significant differences from each other.

**TABLE 5:** Non-inhibitory concentration (NIC) and minimum inhibitory concentration (MIC) values (% v/v) of ethanol obtained for the *S. cerevisiae* strains assayed in this work.

Strains	*NIC* (%v/v)		*MIC* (%v/v)	
	Mean ± SD	CI 95%	Mean ± SD	CI 95%
Calsa	8.69 ± 0.33 C	7.62–9.76	13.73 ± 0.09 B	12.37–15.09
BV37	11.05 ± 0.33 A	10.28–11.82	14.16 ± 0.31 B	13.27–15.05
C73	8.53 ± 0.08 C	7.86–9.19	13.78 ± 0.19 B	12.92–14.64
LC376	7.61 ± 0.24 D	6.15–8.98	14.84 ± 0.23 A	12.71–17.16
Le155	9.48 ± 0.19 B	8.99–9.97	13.55 ± 0.19 B	12.96–14.14
Le384	10.72 ± 0.08 A	10.11–11.33	14.85 ± 0.09 A	14.08–15.61
LF84	10.60 ± 0.08 A	10.09–11.11	13.27 ± 0.31 B	12.69–13.85
LF256	8.91 ± 0.19 C	8.30–9.52	13.51 ± 0.19 B	12.76–14.26
M53	9.40 ± 0.33 B	8.84–9.95	14.97 ± 0.09 A	14.21–15.74
M57	6.32 ± 0.19 E	5.58–7.07	14.13 ± 0.09 B	12.83–15.41
SB31	8.77 ± 0.19 C	8.03–9.51	13.89 ± 0.31 B	12.93–14.84
SR8	9.72 ± 0.09 B	9.25–10.20	14.20 ± 0.09 B	13.61–14.79
SR350	10.44 ± 0.19 A	9.88–11.01	14.09 ± 0.31 B	13.41–14.78
T415	11.00 ± 0.08 A	10.68–11.32	13.68 ± 0.09 B	13.32–14.04

Values are expressed as mean ± standard deviation from triplicate experiment fitted independently. Letters indicate statistically significant groupings determined by the DGC test (*p* < 0.05); strains sharing the same letter within a column do not differ significantly from each other.

**FIGURE 1:**
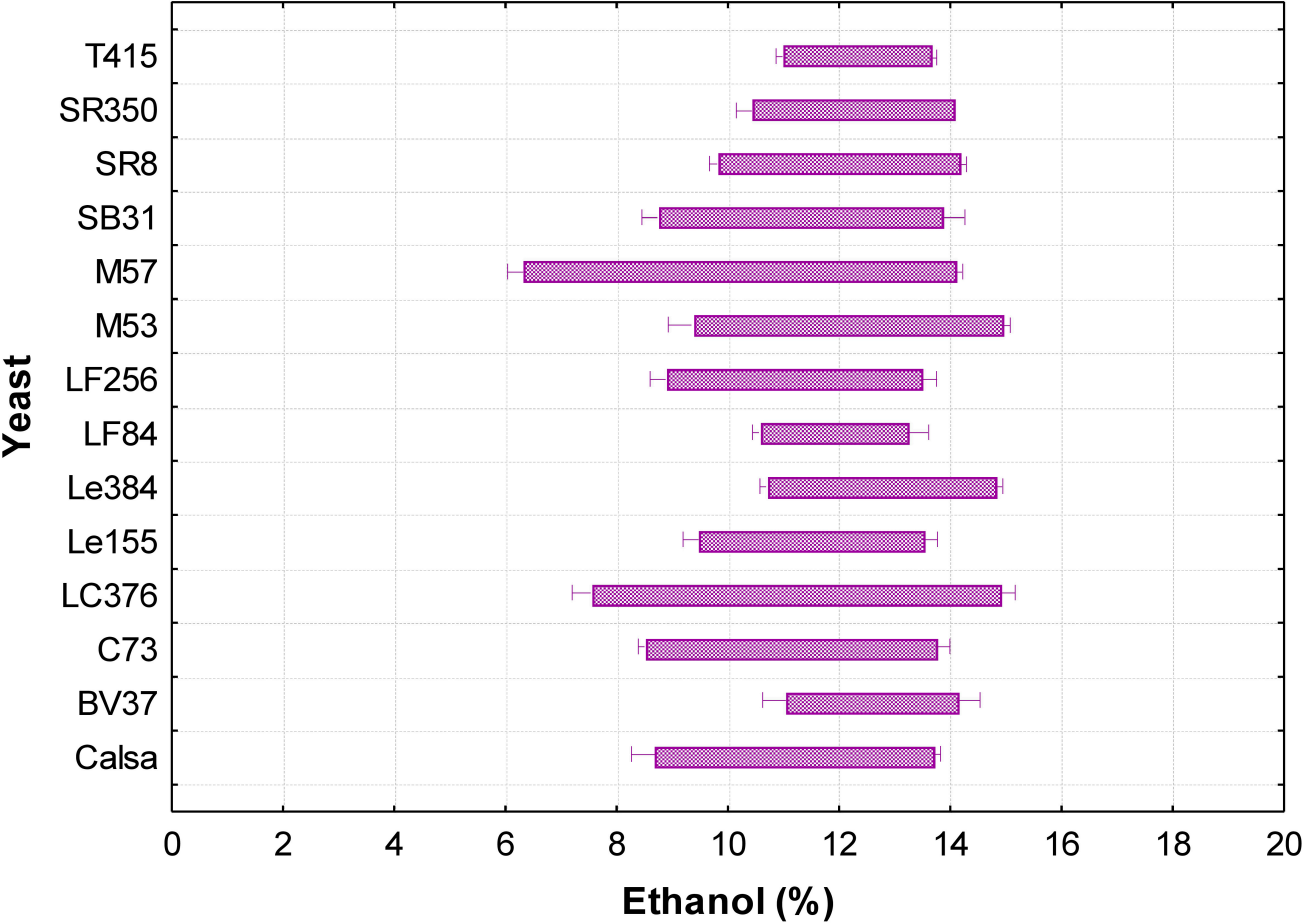
Ethanol inhibition range in *S. cerevisiae* strains. Ethanol concentration ranges (% v/v) between the non-inhibitory concentration (NIC) and the minimum inhibitory concentration (MIC) determined for *S. cerevisiae* strains. This range represents the progressive sensitivity interval to ethanol, where cell growth gradually declines until complete inhibition. Narrower ranges (e.g., Le384) indicate lower initial sensitivity and higher ethanol resistance, while broader ranges (e.g., M57 and LC376) reflect greater adaptability under increasing ethanol concentrations. Strains with lower MIC values, such as LF84 and LF256, show comparatively lower ethanol resistance. Values are averages from triplicate experiments. Lines represent the standard deviations for the different strains.

The range between NIC and MIC was analyzed to evaluate the level of ethanol-induced growth inhibition for each *S. cerevisiae* strain. This range reflects the concentration interval over which growth progressively declines due to increasing ethanol levels, serving as an indicator of both sensitivity and resistance dynamics. As shown in Figure 1, the strain Le384 exhibited the most favorable ethanol tolerance profile, combining a high NIC value (10.72 ± 0.08% v/v) with the highest MIC (14.84 ± 0.23% v/v). Conversely, strains M57 and LC376 exhibited low NIC values; however, their high MIC values (14.13 ± 0.23 and 14.84 ± 0.23% v/v, respectively) indicate that despite early growth inhibition, these strains were able to maintain growth at elevated ethanol concentrations. This results in a broad ethanol inhibition range, characterized by a gradual transition from growth to complete inhibition.

Strains LF84 and T415 showed shorter inhibition intervals with relatively high NIC values but significantly lower MIC values (13.27 ± 0.31 and 13.68 ± 0.09% v/v, respectively), demonstrating reduced ethanol resistance compared to the other strains. Regarding the Calsa strain, it showed a profile similar to that of LF84 and T415, with a comparable MIC (13.73 ± 0.09% v/v) but a slightly lower NIC (8.69 ± 0.33% v/v), suggesting lower overall resistance compared to strains with broader inhibition ranges (Figure 1).

**FIGURE 2:**
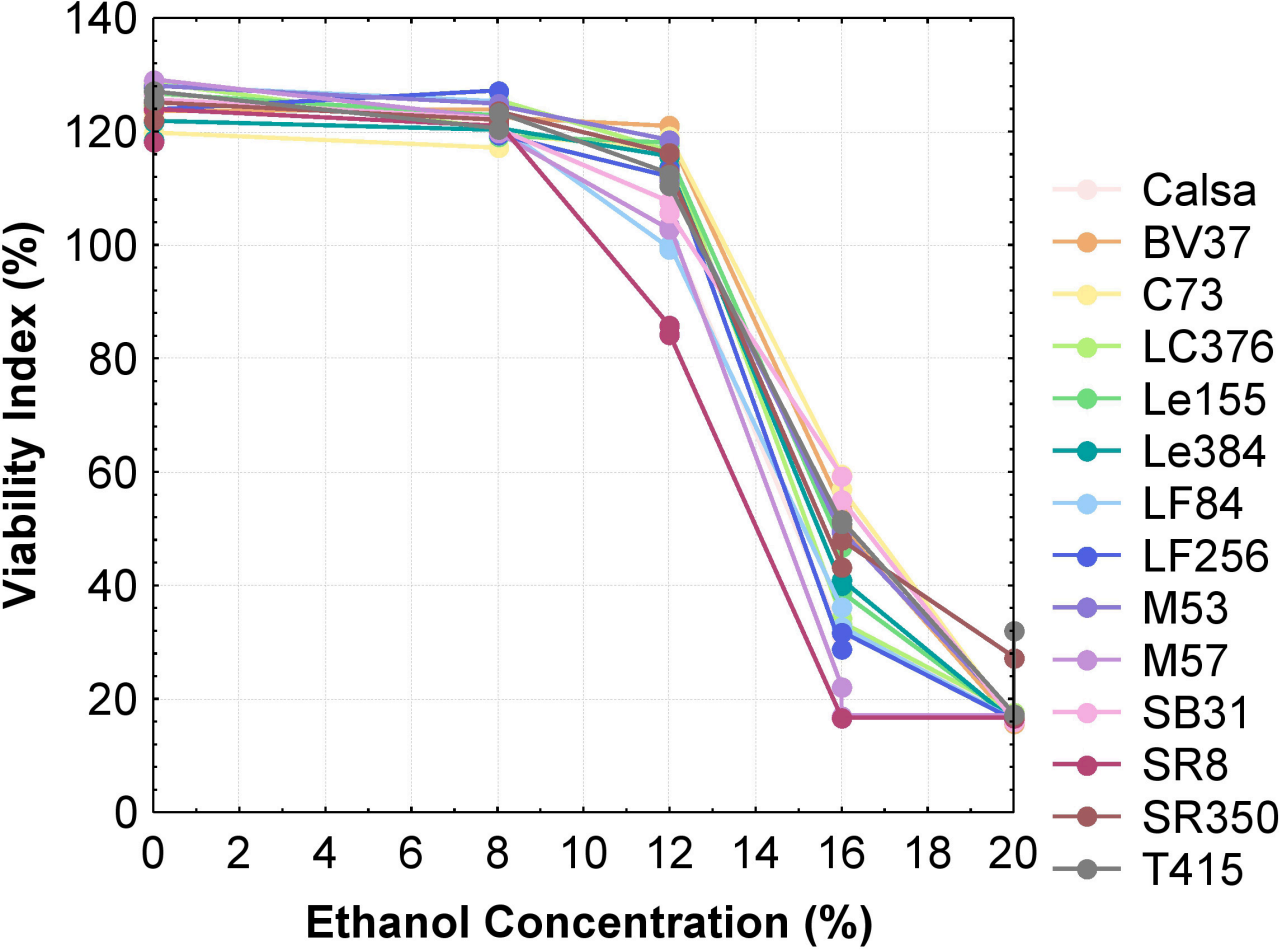
Survival of yeast strains determined by plate count at different ethanol concentrations after 5 days of incubation. Data points represent mean values (*n* = 3) and are expressed as the Viability Index.

To confirm the ethanol tolerance of *S. cerevisiae* strains under prolonged stress conditions by plate count, yeast survival was evaluated after 5 days of incubation in YM broth (pH 5.5) supplemented with increasing ethanol concentrations. Figure 2 displays the survival profiles of the strains exposed to ethanol levels ranging from 0 to 20% v/v. At 0 and 8% v/v ethanol, all strains maintained full viability. In contrast to growth with 8% ethanol, exposure to 12% v/v ethanol led to a moderate but statistically significant reduction in viable cell counts for strains SR8, LF84, M57 and Calsa, with decreases of 36, 23, 18, and 16%, respectively. A marked loss of viability was observed at 16% v/v ethanol across all strains, with survival rates dropping between 17 and 58%. At 20% v/v ethanol, yeast survival was severely compromised, with < 25% of cells remaining viable after 5 days of incubation at 28°C.

## 4 Discussion

The industrial production of bioethanol requires yeast strains with high fermentative efficiency and tolerance to demanding environmental conditions (Canseco Grellet et al., 2022). In this way, the present study applied PM models to characterize native *S. cerevisiae* strains isolated from operating industrial processes. Experiments were conducted under incubation conditions designed to independently assess the main physicochemical factors influencing industrial ethanol fermentation, including temperature, pH, substrate concentration, and ethanol content. Additionally, a commercial reference strain was included for comparison, aiming to identify native variants with equivalent or superior physiological profiles.

Mathematical models such as the CTMI and the CPM were employed to accurately describe the relationship between μ_max_ and temperature or pH conditions. These models, which were validated for their mathematical rigor, have previously demonstrated strong predictive capabilities (Salvadó et al., 2011; Kouamé et al., 2021). In this study, native strains displayed a broad thermal growth range, from 6.3 to 45.6°C, comparable to the best-adapted strains described by Salvadó et al. (2011), who emphasized thermotolerance as a critical adaptive advantage for *S. cerevisiae* in fermentative environments. Among the strains analyzed, LC376, SB31, and T415 demonstrated superior Tmax values, suggesting notable competitive potential under elevated temperature conditions. The use of thermotolerant strains capable of fermenting above 40°C can reduce cooling costs and the risk of microbial contamination in bioprocesses (Shi et al., 2009; Shahsavarani et al., 2012).

Regarding pH, the native strains demonstrated remarkable adaptability across a wide range, from pH 1.93 to 12.93. This behavior is particularly relevant, as pH affects cellular metabolism by altering the ionization state of intracellular metabolites (Ghaffarinasab and Motamedian, 2020). In highly acidic environments, cell membrane stability is compromised, impacting nutrient uptake and tolerance to ethanol accumulation during fermentation (Postaru et al., 2023). These effects may be exacerbated during acid treatments in processes involving cell recycling (Walker and Basso, 2020). Strains SR350 (pH_min_ = 1.93), M57 (2.01), LF84 (2.03), C73 (2.05), Le384 (2.06), SR8 (2.06), T415 (2.06), and M53 (2.07) evidenced ability to grow in markedly acidic conditions, outperforming the industrial control (pH_min_ = 2.13). This level of acid tolerance is necessary in industrial fermentations, where the pH of the must can drop due to the accumulation of organic acids, creating unfavorable conditions for less adapted strains (Liu et al., 2015). These strains exhibited behavior similar to that reported by Liu et al. (2015), who found that certain *S. cerevisiae* strains retained viability and fermentative activity even at pH 2.5. Accordingly, SR350, M57, LF84, C73, Le384, SR8, T415, and M53 are promising candidates for industrial applications requiring high resistance to extreme acidification, such as cell recycling or high-density fermentations.

In assessing sucrose tolerance, strains showed significant variability in their responses to increasing substrate concentrations. The substrate inhibition model proposed by Luong (1987), previously applied successfully to industrial strain characterization (Arroyo-López et al., 2009), enabled estimation of kinetic parameters such as S_opt_ and S_max_ for growth. Notably, maximum sucrose concentrations inducing total growth inhibition (S_max_) in the studied strains far exceeded typical alcoholic fermentation levels, which range from 20 to 25% (w/v) total sugars in the must (Basso et al., 2008). S_max_ values reached up to 73.61%, with optimal growth observed between 3 to 7%, indicating that the responses observed do not directly reflect real-world fermentation conditions and should instead be interpreted within a comparative physiological framework. Thus, while the model displayed strong fit (*R*^2^ > 0.95), it could be particularly useful for evaluating strain suitability during propagation or starter culture preparation stages, where sugar concentrations are generally lower and more controlled (Moutsoglou and Dearden, 2020). In this context, S_opt_ provides practical information on the sucrose concentration that maximizes biomass formation and may be applied when adjusting must dilution during early industrial yeast propagation. S_opt_ and Ks values thus provide useful insights into substrate affinity and growth efficiency, key factors for ensuring successful inoculum establishment at fermentation onset and offer a comparative basis for identifying yeasts with superior sucrose utilization capacity. In addition, these models would allow estimating the behavior in local fermentation systems characterized by different concentrations at the initial fermentation process.

Under ethanol stress Gompertz-based models allowed estimation of NIC and MIC parameters facilitating strain comparison (Lambert and Pearson, 2000; Arroyo-López et al., 2010). Strain Le384 combined a high NIC value (10.72% v/v) with an elevated MIC (14.84% v/v), indicating both low initial sensitivity and high final resistance to ethanol, an advantageous profile for industrial contexts where ethanol concentrations exceed 12% v/v. Similar findings were reported by Kouamé et al. (2021), who also highlighted these models’ utility in predicting strain behavior under multifactorial stress conditions. Besides, findings underscore the importance of considering both the range breadth of the inhibition range and MIC values when selecting strains for ethanol-stress fermentation environments. Additionally, ethanol stress viability index assessments revealed that this strain maintained significant proportions of viable cells even at ethanol concentrations of 16% (v/v). These findings align with those of Arroyo-López et al. (2010), who proposed that ethanol resistance is an ancestral trait, not merely a product of domestication, and may correlate with the lipid composition of cell membranes.

From a methodological perspective, this work integrates primary and secondary growth models to provide a quantitative and predictive characterization of native *S. cerevisiae* strains under multiple stress factors relevant to industrial fermentations. This approach supports more stringent strain selection criteria and reduces experimental workload by enabling the comparison of physiological profiles under controlled conditions (Paula et al., 2019). Taken together, the results establish a robust framework for the pre-selection of promising native yeasts based on growth responses to individual stresses. Validation of ethanol yields and process performance under anaerobic fermentation conditions with cell recycling will be addressed in future pilot- and industrial-scale studies.

## 5 Conclusion

Based on growth-based tolerance parameters, strains T415, Le384, LF84, and SR350 emerged as the most promising candidates relative to the control strain Calsa, particularly regarding ethanol, temperature, and pH stress. These profiles reflect physiological robustness relevant to demanding fermentation environments rather than direct evidence of enhanced ethanol production. Among them, Le384 combined high NIC and MIC values with elevated survival under ethanol exposure and tolerance to acidic pH and high temperatures, whereas T415 showed strong thermal performance and acid resistance. Overall, this study highlights the usefulness of predictive microbiology tools for the technological characterization and pre-selection of native yeasts for bioethanol processes, while fermentation trials remain necessary to confirm industrial performance.

## Data Availability

The original contributions presented in this study are included in this article/Supplementary material, further inquiries can be directed to the corresponding author.

## References

[B1] Arroyo-López F. N. Bautista-Gallego J. Garrido-Fernández A. (2012). “Role of predictive microbiology in food preservation,” in *Progress in Food Preservation*, eds Bhat R. Karim Alias A. Paliyath G. (Hoboken, NJ: Wiley), 10.1002/9781119962045.ch19

[B2] Arroyo-López F. N. Querol A. Barrio E. (2009). Application of a substrate inhibition model to estimate the effect of fructose concentration on the growth of diverse *Saccharomyces cerevisiae* strains. *J. Indus. Microbiol. Biotechnol.* 36 663–669. 10.1007/s10295-009-0535-x 19212785

[B3] Arroyo-López F. N. Salvadoì Z. Tronchoni J. Guillamoìn J. M. Barrio E. Querol A. (2010). Susceptibility and resistance to ethanol in *Saccharomyces* strains isolated from wild and fermentative environments. *Yeast* 27 1005–1015. 10.1002/yea.1809 20824889

[B4] Azhar S. H. M. Abdulla R. Jambo S. A. Marbawi H. Gansau J. A. Faik A. A. M. et al. (2017). Yeasts in sustainable bioethanol production: A review. *Biochem. Biophys. Rep.* 10 52–61. 10.1016/j.bbrep.2017.03.003 29114570 PMC5637245

[B5] Baranyi J. Rockaya M. Ellouze M. (2024). From data to models and predictions in food microbiology. *Curr. Opin. Food Sci.* 57:101177. 10.1016/j.cofs.2024.101177

[B6] Basso C. De Amorim H. V. De Oliveira A. J. Lopes M. L. (2008). Yeast selection for fuel ethanol production in Brazil. *FEMS Yeast Res.* 8 1155–1163. 10.1111/j.1567-1364.2008.00443.x 18752628

[B7] Basso T. O. Walker R. S. K. Basso L. C. Walker G. M. (2019). “The future of bioethanol,” in *Ethanol as a Green Alternative Fuel: Insight and Perspectives*, eds Treichel H. Alves Júnior S. L. Fongaro G. Müller C. (Hauppauge, NY: Nova Science Publishers, Inc), 259–283.

[B8] Canabarro N. I. Silva-Ortiz P. Nogueira L. A. H. Cantarella H. Maciel-Filho R. Souza G. M. (2023). Sustainability assessment of ethanol and biodiesel production in Argentina, Brazil, Colombia, and Guatemala. *Renewable Sustainable Energy Rev.* 171:113019. 10.1016/j.rser.2022.113019

[B9] Canseco Grellet M. A. Dantur K. I. Perera M. F. Ahmed P. M. Castagnaro A. Arroyo-Lopez F. N. et al. (2022). Genotypic and phenotypic characterization of industrial autochthonous *Saccharomyces cerevisiae* for the selection of well-adapted bioethanol-producing strains. *Fungal Biol.* 126 658–673. 10.1016/j.funbio.2022.08.004 36116898

[B10] da Silva Fernández F. de Souza ÉS. Carneiro L. M. Alves Silva J. P. de Souza J. V. B. da Silva Batista J. (2022). Current ethanol production requirements for the yeast Saccharomyces cerevisiae. *Intern. J. Microbiol.* 2022:7878830. 10.1155/2022/7878830 35996633 PMC9392646

[B11] Di Rienzo J. A. Guzmán A. W. Casanoves F. (2002). A multiple-comparisons method based on the distribution of the root node distance of a binary tree. *J. Agricult. Biol. Environ. Statist.* 7 129–142. 10.1198/10857110260141193 12611515

[B12] Favaro L. Jansen T. van Zyl W. H. (2019). Exploring industrial and natural *Saccharomyces cerevisiae* strains for the bio-based economy from biomass: The case of bioethanol. *Crit. Rev. Biotechnol.* 39 800–816. 10.1080/07388551.2019.1619157 31230476

[B13] Ghaffarinasab S. Motamedian E. (2020). Improving ethanol production by studying the effect of pH using a modified metabolic model and a systemic approach. *Biotechnol. Bioeng.* 118 2934–2946. 10.1002/bit.27800 33913513

[B14] Kouamé C. Loiseau G. Grabulos J. Boulanger R. Mestres C. (2021). Development of a model for the alcoholic fermentation of cocoa beans by a *Saccharomyces cerevisiae* strain. *J. Biotechnol.* 326 1–13. 10.1016/j.jbiotec.2020.11.003 33126076

[B15] Kularathne I. W. Ratnaweera A. C. Kalpage C. S. Rajapakse S. Gunathilaka C. A. (2020). Optimization and kinetic parameter estimation of bioethanol production from freely available Sri Lankan fruits in batch fermentation. *Ceylon J. Sci.* 49 283–291. 10.4038/cjs.v49i3.7779

[B16] Kumar D. Singh V. (2016). Dry-grind processing using amylase corn and superior yeast to reduce the exogenous enzyme requirements in bioethanol production. *Biotechnol. Biofuels* 9:228. 10.1186/s13068-016-0649-9 27800014 PMC5078892

[B17] Lambert R. J. Pearson J. (2000). Susceptibility testing: Accurate and reproducible minimum inhibitory concentration (MIC) and non-inhibitory concentration (NIC) values. *J. Appl. Microbiol.* 88 784–790. 10.1046/j.1365-2672.2000.01017.x 10792538

[B18] Liu X. Jia B. Sun X. Ai J. Wang L. Wang C. et al. (2015). Effect of initial ph on growth characteristics and fermentation properties of *Saccharomyces cerevisiae*. *J. Food Sci.* 80 M800–M808. 10.1111/1750-3841.12813 25777552

[B19] Luong J. H. T. (1987). Generalization of Monod kinetics for analysis of growth data with substrate inhibition. *Biotechnol. Bioeng.* 29 242–248. 10.1002/bit.260290215 18576382

[B20] Moutsoglou M. Dearden A. (2020). Efecto del equilibrio respirofermentativo durante la propagación de levaduras en la fermentación y la atenuación del mosto. *J. Instit. Brew.* 126 289–297. 10.1002/jib.621

[B21] Parapouli M. Vasileiadis A. Afendra A. Hatziloukas E. (2020). *Saccharomyces cerevisiae* y sus aplicaciones industriales. *AIMS Microbiol.* 6 1–31. 10.3934/microbiol.2020001 32226912 PMC7099199

[B22] Paula B. P. Chávez D. W. H. Lemos Junior W. J. F. Guerra A. F. Corrêa M. F. D. Pereira K. S. et al. (2019). Growth parameters and survivability of *Saccharomyces boulardii* for probiotic alcoholic beverages development. *Front. Microbiol.* 10:2092. 10.3389/fmicb.2019.02092 31552002 PMC6747048

[B23] Perricone M. Bevilacqua A. Corbo M. R. Sinigaglia M. (2014). Technological characterization and probiotic traits of yeasts isolated from Altamura sourdough to select promising microorganisms as functional starter cultures for cereal-based products. *Food Microbiol.* 38 26–35. 10.1016/j.fm.2013.08.006 24290622

[B24] Petruzzi L. Campaniello D. Corbo M. R. Speranza B. Altieri C. Sinigaglia M. et al. (2022). Wine microbiology and predictive microbiology: A short overview on application, and perspectives. *Microorganisms* 10:421. 10.3390/microorganisms10020421 35208873 PMC8875561

[B25] Postaru M. Tucaliuc A. Cascaval D. Galaction A. I. (2023). Cellular stress impact on yeast activity in biotechnological processes - A short overview. *Microorganisms* 11:2522. 10.3390/microorganisms11102522 37894181 PMC10609598

[B26] Rosso L. Lobry J. R. Flandrois J. P. (1993). An unexpected correlation between cardinal temperatures of microbial growth highlighted by a new model. *J. Theoret. Biol.* 162 447–463. 10.1006/jtbi.1993.1099 8412234

[B27] Rosso L. Lobry J. R. Bajard S. Flandrois J. P. (1995). Convenient model to describe the combined effects of temperature and pH on microbial growth. *Appl. Environ. Microbiol.* 61 610–616. 10.1128/aem.61.2.610-616.1995 16534932 PMC1388350

[B28] Salvadó Z. Arroyo-López F. N. Guillamón J. M. Salazar G. Querol A. Barrio E. (2011). Temperature adaptation markedly determines evolution within the genus Saccharomyces. *Appl. Environ. Microbiol.* 77 2292–2302. 10.1128/AEM.01861-10 21317255 PMC3067424

[B29] Shahsavarani H. Sugiyama M. Kaneko Y. Chuenchit B. Harashima S. (2012). Superior thermotolerance of Saccharomyces cerevisiae for efficient bioethanol fermentation can be achieved by overexpression of RSP5 ubiquitin ligase. *Biotechnol. Adv.* 30 1289–1300. 10.1016/j.biotechadv.2011.09.002 21930195

[B30] Shi D. J. Wang C. L. Wang K. M. (2009). Genome shuffling to improve thermotolerance, ethanol tolerance and ethanol productivity of Saccharomyces cerevisiae. *J. Indus. Microbiol. Biotechnol.* 36 139–147. 10.1007/s10295-008-0481-z 18846398

[B31] Stavropoulou E. Bezirtzoglou E. (2019). Modelado predictivo del comportamiento microbiano en alimentos. *Foods* 8:654. 10.3390/foods8120654 31817788 PMC6963536

[B32] Walker G. M. Basso T. O. (2020). Mitigating stress in industrial yeasts. *Fungal Biol.* 124 387–397. 10.1016/j.funbio.2019.10.010 32389301

[B33] Wani A. K. Rahayu F. Yustina I. Abdul Fatah G. S. Kariada I. K. Antarlina S. S. et al. (2023). Contribution of yeast and its biomass for the preparation of industrially essential materials: A boon to circular economy. *Bioresource Technol. Rep.* 23:101508. 10.1016/j.biteb.2023.101508

[B34] Zhang H. Zhang P. Wu T. Ruan H. (2023). Bioethanol production based on *Saccharomyces cerevisiae*: Opportunities and challenges. *Fermentation* 9:709. 10.3390/fermentation9080709

[B35] Zwietering M. H. Jongenburger I. Rombouts F. M. van ’t Riet K. (1990). Modeling of the bacterial growth curve. *Appl. Environ. Microbiol.* 56 1875–1881. 10.1128/aem.56.6.1875-1881.1990 16348228 PMC184525

