# Supplementary material for: Selection of highly stress-tolerant yeast strains relevant for bioethanol fermentation using predictive growth models
Source: Front Microbiol. 2026 Mar 18;17:1783848. doi: 10.3389/fmicb.2026.1783848 (PMC13038894; doi:10.3389/fmicb.2026.1783848)
Supplement: Supplementary file 1 [file Data_Sheet_1.docx]

# Supplementary Material

**Supplementary Figure 1**. Fit of the Cardinal Temperature Model with Inflection to experimental data (µ_max_) obtained for *S. cerevisiae* Le384 (◆), M57 (■), SB31 (●), and T415 (▲) strains.

**Supplementary Figure 2**. Fit of the Cardinal pH Model to experimental data (µ_max_) obtained for *S. cerevisiae* strains Le384 (◆), M57 (■), SB31 (●), and T415 (▲).

**Supplementary Figure 3**. Area under the growth curves of *S. cerevisiae* strains Le384 (◆), M57 (■), SB31 (●), and T415 (▲) as a function of sucrose concentration, fitted using a generalized Monod-type model.

**Supplementary Figure 4**. Non-inhibitory region (NIR), progressive inhibitory region (PIR), and no-growth region (NGR) identified for *S. cerevisiae* strains Le384 (◆), M57 (■), SB31 (●), and T415 (▲), plotted as a function of the decimal logarithm of ethanol concentration (% v/v) against fa. Curve fitting was performed using a modified Gompertz decay model (Lambert and Pearson, 2000).

**Supplementary Table 1.** Growth parameters for autochthonous and control *S. cerevisiae* strains as a function of temperature estimated using the reparameterized Gompertz primary model equation.

|  |  | **D** | | **µ_max_ (h^-1^)** | | **λ (h)** | |
| --- | --- | --- | --- | --- | --- | --- | --- |
|  |  | mean ± SD | CI 95 % | mean ± SD | CI 95 % | mean ± SD | CI 95 % |
| Calsa | 4 | NG |  | NG |  | NG |  |
|  | 10 | 1.32 ± 0.05 | 1.29 - 1.35 | 0.02 ± 0.00 | 0.02 - 0.03 | 19.86 ± 2.34 | 16.33 - 23.40 |
|  | 16 | 1.35 ± 0.08 | 1.29 - 1.42 | 0.04 ± 0.01 | 0.03 - 0.05 | 2.87 ± 2.79 | 0.00 - 9.46 |
|  | 22 | 1.26 ± 0.06 | 1.24 - 1.27 | 0.12 ± 0.01 | 0.11 - 0.13 | 4.93 ± 0.79 | 4.28 - 5.57 |
|  | 28 | 1.27 ± 0.03 | 1.26 - 1.28 | 0.21 ± 0.02 | 0.19 - 0.23 | 3.08 ± 0.27 | 2.78 - 3.37 |
|  | 32 | 1.26 ± 0.01 | 1.25 - 1.27 | 0.20 ± 0.01 | 0.18 - 0.22 | 2.25 ± 0.19 | 1.87 - 2.63 |
|  | 36 | 1.31 ± 0.04 | 1.30 - 1.32 | 0.23 ± 0.01 | 0.20 - 0.26 | 0.88 ± 0.51 | 0.48 - 1.28 |
|  | 40 | NG |  | NG |  | NG |  |
|  | 43 | NG |  | NG |  | NG |  |
|  | 46 | NG |  | NG |  | NG |  |
| BV37 | 4 | NG |  | NG |  | NG |  |
|  | 10 | 1.31 ± 0.02 | 1.28 - 1.35 | 0.02 ± 0.00 | 0.02 - 0.03 | 18.52 ± 3.12 | 14.50 - 22.55 |
|  | 16 | 1.42 ± 0.01 | 1.37 - 1.47 | 0.06 ± 0.01 | 0.05 - 0.08 | 6.33 ± 0.40 | 2.59 - 10.06 |
|  | 22 | 1.24 ± 0.02 | 1.22 - 1.25 | 0.12 ± 0.00 | 0.10 - 0.13 | 4.03 ± 0.21 | 3.31 - 4.75 |
|  | 28 | 1.25 ± 0.06 | 1.24 - 1.25 | 0.21 ± 0.01 | 0.19 - 0.23 | 1.59 ± 0.73 | 1.33 - 1.86 |
|  | 32 | 1.40 ± 0.01 | 1.39 - 1.41 | 0.23 ± 0.00 | 0.21 - 0.26 | 1.35 ± 0.01 | 1.05 - 1.66 |
|  | 36 | 1.32 ± 0.02 | 1.31 - 1.32 | 0.23 ± 0.01 | 0.20 - 0.26 | 1.95 ± 0.43 | 1.59 - 2.30 |
|  | 40 | 0.89 ± 0.04 | 0.87 - 0.92 | 0.02 ± 0.00 | 0.02 - 0.03 | 3.01 ± 0.50 | 0.00 - 7.23 |
|  | 43 | NG |  | NG |  | NG |  |
|  | 46 | NG |  | NG |  | NG |  |
| C73 | 4 | 1.53 ± 0.20 | 0.77 - 2.28 | 0.00 ± 0.00 | 0.00 - 0.01 | 33.16 ± 20.50 | 0.44 - 65.88 |
|  | 10 | 1.60 ± 0.08 | 1.56 - 1.63 | 0.03 ± 0.00 | 0.02 - 0.03 | 19.89 ± 4.18 | 16.25 - 23.53 |
|  | 16 | 1.57 ± 0.03 | 1.54 - 1.61 | 0.08 ± 0.01 | 0.06 - 0.09 | 8.38 ± 0.57 | 5.61 - 11.15 |
|  | 22 | 1.41 ± 0.00 | 1.40 - 1.42 | 0.15 ± 0.00 | 0.14 - 0.17 | 5.49 ± 0.10 | 5.00 - 5.98 |
|  | 28 | 1.38 ± 0.01 | 1.38 - 1.39 | 0.19 ± 0.00 | 0.17 - 0.20 | 1.83 ± 0.08 | 1.49 - 2.17 |
|  | 32 | 1.49 ± 0.01 | 1.49 - 1.50 | 0.26 ± 0.02 | 0.24 - 0.28 | 2.12 ± 0.26 | 1.85 - 2.38 |
|  | 36 | 1.43 ± 0.01 | 1.43 - 1.44 | 0.24 ± 0.00 | 0.22 - 0.26 | 2.38 ± 0.79 | 2.14 - 2.63 |
|  | 40 | 0.84 ± 0.17 | 0.82 - 0.87 | 0.02 ± 0.00 | 0.02 - 0.03 | 1.93 ± 1.42 | 0.00 - 6.11 |
|  | 43 | NG |  | NG |  | NG |  |
|  | 46 | NG |  | NG |  | NG |  |
| LC376 | 4 | NG |  | NG |  | NG |  |
|  | 10 | 1.48 ± 0.02 | 1.44 - 1.51 | 0.02 ± 0.00 | 0.02 - 0.02 | 14.32 ± 1.57 | 10.74 - 17.89 |
|  | 16 | 1.31 ± 0.09 | 1.27 - 1.34 | 0.06 ± 0.02 | 0.04 - 0.08 | 11.11 ± 3.76 | 7.24 - 14.99 |
|  | 22 | 1.23 ± 0.02 | 1.22 - 1.24 | 0.10 ± 0.00 | 0.10 - 0.11 | 4.84 ± 0.53 | 4.29 - 5.38 |
|  | 28 | 1.15 ± 0.03 | 1.15 - 1.16 | 0.14 ± 0.01 | 0.13 - 0.15 | 2.65 ± 0.22 | 2.28 - 3.01 |
|  | 32 | 1.34 ± 0.03 | 1.33 - 1.35 | 0.18 ± 0.01 | 0.16 - 0.20 | 1.48 ± 0.13 | 1.09 - 1.87 |
|  | 36 | 1.28 ± 0.02 | 1.27 - 1.29 | 0.20 ± 0.00 | 0.18 - 0.22 | 2.13 ± 0.11 | 1.83 - 2.44 |
|  | 40 | NG |  | NG |  | NG |  |
|  | 43 | NG |  | NG |  | NG |  |
|  | 46 | NG |  | NG |  | NG |  |
| Le155 | 4 | NG |  | NG |  | NG |  |
|  | 10 | 1.47 ± 0.07 | 1.43 - 1.51 | 0.03 ± 0.00 | 0.02 - 0.03 | 18.03 ± 0.85 | 14.02 - 22.04 |
|  | 16 | 1.20 ± 0.07 | 1.15 - 1.25 | 0.05 ± 0.01 | 0.03 - 0.07 | 7.36 ± 1.21 | 2.30 - 12.42 |
|  | 22 | 1.30 ± 0.03 | 1.29 - 1.31 | 0.13 ± 0.00 | 0.11 - 0.14 | 3.76 ± 0.18 | 3.11 - 4.41 |
|  | 28 | 1.26 ± 0.01 | 1.25 - 1.27 | 0.19 ± 0.00 | 0.17 - 0.21 | 0.72 ± 0.01 | 0.36 - 1.08 |
|  | 32 | 1.37 ± 0.02 | 1.36 - 1.38 | 0.22 ± 0.00 | 0.20 - 0.25 | 1.30 ± 0.08 | 0.95 - 1.64 |
|  | 36 | 1.35 ± 0.04 | 1.34 - 1.35 | 0.26 ± 0.01 | 0.23 - 0.28 | 1.33 ± 0.16 | 1.04 - 1.61 |
|  | 40 | NG |  | NG |  | NG |  |
|  | 43 | NG |  | NG |  | NG |  |
|  | 46 | NG |  | NG |  | NG |  |
| Le384 | 4 | NG |  | NG |  | NG |  |
|  | 10 | 1.55 ± 0.09 | 1.50 - 1.59 | 0.03 ± 0.00 | 0.02 - 0.03 | 18.62 ± 3.60 | 14.24 - 22.99 |
|  | 16 | 1.41 ± 0.02 | 1.36 - 1.46 | 0.05 ± 0.01 | 0.04 - 0.07 | 7.75 ± 0.70 | 2.72 - 12.78 |
|  | 22 | 1.43 ± 0.06 | 1.42 - 1.44 | 0.13 ± 0.02 | 0.12 - 0.14 | 4.13 ± 1.16 | 3.63 - 4.63 |
|  | 28 | 1.37 ± 0.01 | 1.36 - 1.38 | 0.18 ± 0.00 | 0.17 - 0.20 | 0.11 ± 0.10 | 0.00 - 0.48 |
|  | 32 | 1.47 ± 0.03 | 1.46 - 1.48 | 0.23 ± 0.01 | 0.20 - 0.25 | 1.73 ± 0.04 | 1.33 - 2.13 |
|  | 36 | 1.27 ± 0.04 | 1.26 - 1.29 | 0.18 ± 0.03 | 0.15 - 0.20 | 1.64 ± 0.22 | 1.04 - 2.24 |
|  | 40 | 0.92 ± 0.04 | 0.89 - 0.95 | 0.02 ± 0.00 | 0.02 - 0.03 | 6.56 ± 0.11 | 2.65 - 10.48 |
|  | 43 | NG |  | NG |  | NG |  |
|  | 46 | NG |  | NG |  | NG |  |
| LF84 | 4 | 1.74 ± 0.19 | 0.85 - 2.64 | 0.00 ± 0.00 | 0.00 - 0.00 | 47.94 ± 18.15 | 17.19 - 78.70 |
|  | 10 | 1.54 ± 0.05 | 1.50 - 1.57 | 0.03 ± 0.00 | 0.03 - 0.04 | 17.09 ± 0.44 | 13.70 - 20.47 |
|  | 16 | 1.33 ± 0.07 | 1.29 - 1.38 | 0.04 ± 0.01 | 0.03 - 0.05 | 5.46 ± 0.32 | 0.69 - 10.23 |
|  | 22 | 1.36 ± 0.02 | 1.35 - 1.37 | 0.14 ± 0.00 | 0.13 - 0.15 | 2.84 ± 0.23 | 2.38 - 3.30 |
|  | 28 | 1.21 ± 0.01 | 1.20 - 1.21 | 0.17 ± 0.03 | 0.16 - 0.19 | 0.89 ± 0.07 | 0.54 - 1.25 |
|  | 32 | 1.40 ± 0.01 | 1.39 - 1.41 | 0.26 ± 0.01 | 0.24 - 0.29 | 1.61 ± 0.18 | 1.31 - 1.91 |
|  | 36 | 1.26 ± 0.01 | 1.25 - 1.26 | 0.26 ± 0.00 | 0.24 - 0.28 | 1.61 ± 0.06 | 1.37 - 1.85 |
|  | 40 | NG |  | NG |  | NG |  |
|  | 43 | NG |  | NG |  | NG |  |
|  | 46 | NG |  | NG |  | NG |  |
| LF256 | 4 | NG |  | NG |  | NG |  |
|  | 10 | 1.60 ± 0.05 | 1.55 - 1.65 | 0.03 ± 0.00 | 0.02 - 0.03 | 18.42 ± 1.64 | 13.60 - 23.23 |
|  | 16 | 1.48 ± 0.03 | 1.45 - 1.51 | 0.07 ± 0.00 | 0.06 - 0.08 | 8.16 ± 0.32 | 5.77 - 10.55 |
|  | 22 | 1.43 ± 0.01 | 1.42 - 1.44 | 0.15 ± 0.00 | 0.14 - 0.16 | 3.21 ± 0.26 | 2.83 - 3.59 |
|  | 28 | 1.33 ± 0.01 | 1.32 - 1.33 | 0.18 ± 0.00 | 0.17 - 0.20 | 2.18 ± 0.47 | 1.88 - 2.48 |
|  | 32 | 1.47 ± 0.04 | 1.46 - 1.47 | 0.25 ± 0.01 | 0.23 - 0.27 | 1.75 ± 0.22 | 1.49 - 2.02 |
|  | 36 | 1.46 ± 0.01 | 1.45 - 1.46 | 0.25 ± 0.00 | 0.23 - 0.26 | 1.52 ± 0.18 | 1.29 - 1.75 |
|  | 40 | 1.00 ± 0.05 | 0.97 - 1.03 | 0.03 ± 0.00 | 0.02 - 0.03 | 2.49 ± 0.36 | 0.00 - 6.34 |
|  | 43 | NG |  | NG |  | NG |  |
|  | 46 | NG |  | NG |  | NG |  |
| M53 | 4 | NG |  | NG |  | NG |  |
|  | 10 | 1.67 ± 0.05 | 1.63 - 1.71 | 0.03 ± 0.00 | 0.02 - 0.03 | 15.00 ± 1.09 | 11.21 - 18.79 |
|  | 16 | 1.36 ± 0.09 | 1.33 - 1.40 | 0.05 ± 0.01 | 0.04 - 0.06 | 9.33 ± 0.69 | 5.79 - 12.87 |
|  | 22 | 1.45 ± 0.01 | 1.45 - 1.46 | 0.13 ± 0.00 | 0.12 - 0.14 | 2.65 ± 0.02 | 2.28 - 3.03 |
|  | 28 | 1.37 ± 0.03 | 1.37 - 1.38 | 0.19 ± 0.01 | 0.18 - 0.21 | 1.50 ± 0.30 | 1.21 - 1.79 |
|  | 32 | 1.42 ± 0.02 | 1.41 - 1.43 | 0.21 ± 0.01 | 0.20 - 0.23 | 1.71 ± 0.16 | 1.41 - 2.01 |
|  | 36 | 1.28 ± 0.09 | 1.27 - 1.28 | 0.18 ± 0.02 | 0.17 - 0.19 | 2.33 ± 0.48 | 2.07 - 2.59 |
|  | 40 | 0.77 ± 0.16 | 0.74 - 0.80 | 0.02 ± 0.01 | 0.01 - 0.02 | 6.63 ± 1.36 | 1.60 - 11.65 |
|  | 43 | NG |  | NG |  | NG |  |
|  | 46 | NG |  | NG |  | NG |  |
| M57 | 4 | NG |  | NG |  | NG |  |
|  | 10 | 0.91 ± 0.18 | 0.89 - 0.94 | 0.01 ± 0.00 | 0.01 - 0.01 | 5.85 ± 3.39 | 0.28 - 11.42 |
|  | 16 | 1.05 ± 0.12 | 1.03 - 1.07 | 0.03 ± 0.00 | 0.02 - 0.03 | 13.54 ± 3.35 | 11.19 - 15.90 |
|  | 22 | 1.37 ± 0.02 | 1.36 - 1.38 | 0.09 ± 0.00 | 0.09 - 0.10 | 4.64 ± 0.17 | 4.10 - 5.17 |
|  | 28 | 1.27 ± 0.04 | 1.27 - 1.28 | 0.13 ± 0.01 | 0.12 - 0.14 | 1.83 ± 0.23 | 1.38 - 2.29 |
|  | 32 | 1.37 ± 0.03 | 1.36 - 1.38 | 0.17 ± 0.01 | 0.15 - 0.19 | 2.82 ± 0.17 | 2.35 - 3.30 |
|  | 36 | 1.15 ± 0.07 | 1.14 - 1.15 | 0.15 ± 0.02 | 0.14 - 0.16 | 3.50 ± 0.12 | 3.15 - 3.85 |
|  | 40 | 0.49 ± 0.02 | 0.47 - 0.51 | 0.01 ± 0.00 | 0.01 - 0.01 | 11.57 ± 3.24 | 6.11 - 17.03 |
|  | 43 | NG |  | NG |  | NG |  |
|  | 46 | NG |  | NG |  | NG |  |
| SB31 | 4 | NG |  | NG |  | NG |  |
|  | 10 | 1.53 ± 0.02 | 1.47 - 1.59 | 0.02 ± 0.00 | 0.02 - 0.03 | 9.09 ± 0.21 | 2.66 - 15.51 |
|  | 16 | 1.46 ± 0.05 | 1.42 - 1.49 | 0.06 ± 0.01 | 0.05 - 0.07 | 6.64 ± 1.93 | 3.65 - 9.63 |
|  | 22 | 1.41 ± 0.01 | 1.40 - 1.42 | 0.15 ± 0.01 | 0.14 - 0.16 | 3.51 ± 0.11 | 3.10 - 3.93 |
|  | 28 | 1.27 ± 0.01 | 1.26 - 1.27 | 0.19 ± 0.00 | 0.17 - 0.20 | 1.39 ± 0.07 | 1.08 - 1.71 |
|  | 32 | 1.47 ± 0.02 | 1.46 - 1.47 | 0.26 ± 0.01 | 0.24 - 0.28 | 1.70 ± 0.16 | 1.46 - 1.93 |
|  | 36 | 1.43 ± 0.05 | 1.43 - 1.44 | 0.27 ± 0.01 | 0.25 - 0.29 | 1.60 ± 0.20 | 1.38 - 1.82 |
|  | 40 | 1.17 ± 0.00 | 1.14 - 1.20 | 0.03 ± 0.00 | 0.03 - 0.04 | 2.29 ± 0.55 | 0.00 - 5.76 |
|  | 43 | NG |  | NG |  | NG |  |
|  | 46 | NG |  | NG |  | NG |  |
| SR8 | 4 | NG |  | NG |  | NG |  |
|  | 10 | 1.55 ± 0.09 | 1.50 - 1.59 | 0.03 ± 0.00 | 0.02 - 0.03 | 18.59 ± 3.60 | 14.16 - 23.01 |
|  | 16 | 1.41 ± 0.02 | 1.36 - 1.46 | 0.05 ± 0.01 | 0.04 - 0.07 | 7.72 ± 0.70 | 2.69 - 12.75 |
|  | 22 | 1.43 ± 0.06 | 1.42 - 1.44 | 0.13 ± 0.02 | 0.12 - 0.14 | 4.12 ± 1.17 | 3.61 - 4.64 |
|  | 28 | 1.37 ± 0.01 | 1.36 - 1.38 | 0.18 ± 0.01 | 0.17 - 0.20 | 0.13 ± 0.14 | 0.00 - 0.50 |
|  | 32 | 1.47 ± 0.03 | 1.46 - 1.48 | 0.23 ± 0.01 | 0.20 - 0.25 | 1.71 ± 0.04 | 1.30 - 2.13 |
|  | 36 | 1.27 ± 0.04 | 1.26 - 1.29 | 0.18 ± 0.03 | 0.15 - 0.20 | 1.67 ± 0.20 | 1.08 - 2.26 |
|  | 40 | NG |  | NG |  | NG |  |
|  | 43 | NG |  | NG |  | NG |  |
|  | 46 | NG |  | NG |  | NG |  |
| SR350 | 4 | NG |  | NG |  | NG |  |
|  | 10 | 1.41 ± 0.06 | 1.37 - 1.45 | 0.02 ± 0.00 | 0.02 - 0.03 | 14.15 ± 1.58 | 9.53 - 18.78 |
|  | 16 | 1.26 ± 0.05 | 1.23 - 1.29 | 0.06 ± 0.00 | 0.04 - 0.07 | 10.31 ± 1.53 | 7.09 - 13.53 |
|  | 22 | 1.34 ± 0.03 | 1.33 - 1.35 | 0.12 ± 0.00 | 0.12 - 0.13 | 3.64 ± 0.36 | 3.18 - 4.10 |
|  | 28 | 1.33 ± 0.03 | 1.32 - 1.33 | 0.23 ± 0.00 | 0.21 - 0.24 | 1.05 ± 0.16 | 0.81 - 1.29 |
|  | 32 | 1.36 ± 0.05 | 1.35 - 1.36 | 0.22 ± 0.01 | 0.19 - 0.24 | 1.75 ± 0.14 | 1.37 - 2.13 |
|  | 36 | 1.33 ± 0.01 | 1.32 - 1.33 | 0.23 ± 0.00 | 0.21 - 0.26 | 2.03 ± 0.05 | 1.70 - 2.35 |
|  | 40 | NG |  | NG |  | NG |  |
|  | 43 | NG |  | NG |  | NG |  |
|  | 46 | NG |  | NG |  | NG |  |
| T415 | 4 | NG |  | NG |  | NG |  |
|  | 10 | 1.60 ± 0.04 | 1.57 - 1.63 | 0.02 ± 0.00 | 0.02 - 0.03 | 15.16 ± 2.28 | 11.70 - 18.62 |
|  | 16 | 1.53 ± 0.05 | 1.49 - 1.57 | 0.06 ± 0.01 | 0.05 - 0.07 | 7.81 ± 0.13 | 4.43 - 11.19 |
|  | 22 | 1.40 ± 0.02 | 1.39 - 1.41 | 0.13 ± 0.00 | 0.12 - 0.14 | 3.20 ± 0.19 | 2.76 - 3.63 |
|  | 28 | 1.31 ± 0.02 | 1.31 - 1.32 | 0.19 ± 0.00 | 0.18 - 0.20 | 1.05 ± 0.18 | 0.81 - 1.30 |
|  | 32 | 1.49 ± 0.02 | 1.48 - 1.49 | 0.22 ± 0.01 | 0.20 - 0.24 | 1.37 ± 0.08 | 1.02 - 1.72 |
|  | 36 | 1.41 ± 0.02 | 1.39 - 1.42 | 0.19 ± 0.03 | 0.16 - 0.21 | 1.26 ± 0.14 | 0.68 - 1.83 |
|  | 40 | 0.54 ± 0.04 | 0.53 - 0.56 | 0.01 ± 0.00 | 0.01 - 0.02 | 1.63 ± 2.38 | 0.00 - 5.89 |
|  | 43 | NG |  | NG |  | NG |  |
|  | 46 | NG |  | NG |  | NG |  |

Parameters: *D* is the maximum OD reached; µ_max_ denotes the maximum specific growth rate (h^-1^); and λ the lag phase period (h). Values represent the mean ± standard deviation from three independent replicates. NG: no growth.

**Supplementary Table 2**. Growth parameters for autochthonous and control *S. cerevisiae* strains as a function of pH estimated using the reparameterized Gompertz primary model equation.

|  |  | **D** | | **µ_max_ (h^-1^)** | | **λ (h)** | |
| --- | --- | --- | --- | --- | --- | --- | --- |
|  |  | mean ± SD | CI 95 % | mean ± SD | CI 95 % | mean ± SD | CI 95 % |
| Calsa | 2 | NG |  | NG |  | NG |  |
|  | 2.5 | NG |  | NG |  | NG |  |
|  | 3 | 1.12 ± 0.08 | 1.11 - 1.13 | 0.11 ± 0.01 | 0.10 - 0.12 | 2.24 ± 0.11 | 1.69 - 2.80 |
|  | 4.5 | 1.63 ± 0.02 | 1.60 - 1.66 | 0.17 ± 0.01 | 0.13 - 0.21 | 1.48 ± 0.17 | 0.41 - 2.54 |
|  | 5.5 | 1.56 ± 0.13 | 1.54 - 1.59 | 0.18 ± 0.01 | 0.14 - 0.22 | 1.90 ± 0.44 | 0.91 - 2.89 |
|  | 8.5 | 1.51 ± 0.14 | 1.48 - 1.53 | 0.16 ± 0.04 | 0.13 - 0.20 | 2.20 ± 0.70 | 1.06 - 3.34 |
|  | 11 | NG |  | NG |  | NG |  |
|  | 11.5 | NG |  | NG |  | NG |  |
|  | 12 | NG |  | NG |  | NG |  |
| BV37 | 2 | NG |  | NG |  | NG |  |
|  | 2.5 | 0.41 ± 0.06 | 0.41 - 0.42 | 0.01 ± 0.00 | 0.01 - 0.01 | 0.53 ± 0.75 | 0.00 - 2.01 |
|  | 3 | 1.51 ± 0.19 | 1.50 - 1.53 | 0.13 ± 0.02 | 0.11 - 0.14 | 2.73 ± 0.90 | 2.04 - 3.42 |
|  | 4.5 | 1.61 ± 0.03 | 1.59 - 1.63 | 0.20 ± 0.00 | 0.17 - 0.23 | 1.35 ± 0.31 | 0.76 - 1.95 |
|  | 5.5 | 1.61 ± 0.05 | 1.58 - 1.63 | 0.23 ± 0.03 | 0.19 - 0.27 | 1.07 ± 0.32 | 0.46 - 1.68 |
|  | 8.5 | 1.56 ± 0.05 | 1.54 - 1.59 | 0.20 ± 0.00 | 0.16 - 0.24 | 1.64 ± 0.12 | 0.80 - 2.48 |
|  | 11 | NG |  | NG |  | NG |  |
|  | 11.5 | NG |  | NG |  | NG |  |
|  | 12 | NG |  | NG |  | NG |  |
| C73 | 2 | NG |  | NG |  | NG |  |
|  | 2.5 | 0.52 ± 0.06 | 0.52 - 0.53 | 0.01 ± 0.00 | 0.01 - 0.02 | 4.09 ± 0.18 | 2.80 - 5.37 |
|  | 3 | 1.70 ± 0.08 | 1.68 - 1.72 | 0.11 ± 0.00 | 0.10 - 0.12 | 1.70 ± 0.64 | 0.82 - 2.58 |
|  | 4.5 | 1.79 ± 0.04 | 1.76 - 1.81 | 0.20 ± 0.02 | 0.17 - 0.23 | 1.31 ± 0.11 | 0.63 - 1.99 |
|  | 5.5 | 1.75 ± 0.03 | 1.72 - 1.77 | 0.20 ± 0.01 | 0.16 - 0.24 | 1.82 ± 0.12 | 0.98 - 2.66 |
|  | 8.5 | 1.59 ± 0.11 | 1.56 - 1.63 | 0.17 ± 0.05 | 0.12 - 0.21 | 1.27 ± 1.01 | 0.00 - 2.57 |
|  | 11 | NG |  | NG |  | NG |  |
|  | 11.5 | NG |  | NG |  | NG |  |
|  | 12 | NG |  | NG |  | NG |  |
| LC376 | 2 | NG |  | NG |  | NG |  |
|  | 2.5 | NG |  | NG |  | NG |  |
|  | 3 | 1.52 ± 0.29 | 1.51 - 1.54 | 0.11 ± 0.00 | 0.10 - 0.13 | 2.48 ± 0.51 | 1.81 - 3.15 |
|  | 4.5 | 1.63 ± 0.01 | 1.61 - 1.65 | 0.20 ± 0.01 | 0.17 - 0.23 | 1.17 ± 0.08 | 0.49 - 1.84 |
|  | 5.5 | 1.55 ± 0.02 | 1.53 - 1.56 | 0.23 ± 0.01 | 0.20 - 0.26 | 1.41 ± 0.41 | 0.92 - 1.90 |
|  | 8.5 | 1.88 ± 0.30 | 1.86 - 1.90 | 0.20 ± 0.01 | 0.17 - 0.23 | 1.79 ± 0.19 | 1.13 - 2.44 |
|  | 11 | NG |  | NG |  | NG |  |
|  | 11.5 | NG |  | NG |  | NG |  |
|  | 12 | NG |  | NG |  | NG |  |
| Le155 | 2 | NG |  | NG |  | NG |  |
|  | 2.5 | 0.54 ± 0.02 | 0.51 - 0.57 | 0.01 ± 0.00 | 0.01 - 0.01 | 0.11 ± 3.89 | 0.00 - 5.05 |
|  | 3 | 1.43 ± 0.05 | 1.43 - 1.44 | 0.13 ± 0.00 | 0.12 - 0.14 | 2.77 ± 0.36 | 2.40 - 3.13 |
|  | 4.5 | 1.46 ± 0.06 | 1.45 - 1.47 | 0.22 ± 0.01 | 0.20 - 0.24 | 1.41 ± 0.11 | 1.09 - 1.72 |
|  | 5.5 | 1.62 ± 0.04 | 1.61 - 1.64 | 0.23 ± 0.02 | 0.20 - 0.27 | 1.46 ± 0.08 | 0.96 - 1.96 |
|  | 8.5 | 1.58 ± 0.01 | 1.56 - 1.59 | 0.20 ± 0.02 | 0.17 - 0.23 | 1.73 ± 0.05 | 1.16 - 2.30 |
|  | 11 | NG |  | NG |  | NG |  |
|  | 11.5 | NG |  | NG |  | NG |  |
|  | 12 | NG |  | NG |  | NG |  |
| Le384 | 2 | NG |  | NG |  | NG |  |
|  | 2.5 | 1.15 ± 0.04 | 1.14 - 1.17 | 0.04 ± 0.01 | 0.03 - 0.04 | 4.52 ± 1.68 | 3.30 - 5.75 |
|  | 3 | 1.37 ± 0.04 | 1.35 - 1.38 | 0.14 ± 0.01 | 0.12 - 0.15 | 1.99 ± 0.25 | 1.40 - 2.57 |
|  | 4.5 | 1.54 ± 0.01 | 1.53 - 1.55 | 0.20 ± 0.02 | 0.18 - 0.21 | 1.45 ± 0.25 | 1.10 - 1.80 |
|  | 5.5 | 1.74 ± 0.04 | 1.72 - 1.77 | 0.17 ± 0.01 | 0.14 - 0.19 | 0.88 ± 0.11 | 0.07 - 1.69 |
|  | 8.5 | 1.64 ± 0.07 | 1.62 - 1.67 | 0.12 ± 0.03 | 0.10 - 0.14 | 1.70 ± 0.97 | 0.47 - 2.93 |
|  | 11 | NG |  | NG |  | NG |  |
|  | 11.5 | NG |  | NG |  | NG |  |
|  | 12 | NG |  | NG |  | NG |  |
| LF84 | 2 | NG |  | NG |  | NG |  |
|  | 2.5 | NG |  | NG |  | NG |  |
|  | 3 | 1.65 ± 0.02 | 1.64 - 1.66 | 0.13 ± 0.00 | 0.12 - 0.14 | 3.32 ± 0.15 | 2.92 - 3.73 |
|  | 4.5 | 1.49 ± 0.03 | 1.48 - 1.51 | 0.18 ± 0.01 | 0.16 - 0.21 | 1.36 ± 0.23 | 0.82 - 1.91 |
|  | 5.5 | 1.55 ± 0.06 | 1.54 - 1.57 | 0.21 ± 0.00 | 0.18 - 0.23 | 1.33 ± 0.05 | 0.87 - 1.78 |
|  | 8.5 | 1.39 ± 0.04 | 1.37 - 1.40 | 0.19 ± 0.01 | 0.16 - 0.22 | 2.30 ± 0.35 | 1.62 - 2.98 |
|  | 11 | NG |  | NG |  | NG |  |
|  | 11.5 | NG |  | NG |  | NG |  |
|  | 12 | NG |  | NG |  | NG |  |
| LF256 | 2 | NG |  | NG |  | NG |  |
|  | 2.5 | 0.86 ± 0.23 | 0.84 - 0.87 | 0.02 ± 0.01 | 0.01 - 0.02 | 0.56 ± 1.93 | 0.00 - 2.29 |
|  | 3 | 1.43 ± 0.04 | 1.41 - 1.44 | 0.11 ± 0.00 | 0.10 - 0.12 | 2.36 ± 0.21 | 1.71 - 3.01 |
|  | 4.5 | 1.72 ± 0.01 | 1.70 - 1.74 | 0.20 ± 0.01 | 0.17 - 0.23 | 2.05 ± 0.06 | 1.32 - 2.79 |
|  | 5.5 | 1.62 ± 0.03 | 1.60 - 1.63 | 0.19 ± 0.01 | 0.17 - 0.22 | 1.86 ± 0.32 | 1.25 - 2.46 |
|  | 8.5 | 1.00 ± 0.04 | 0.98 - 1.02 | 0.07 ± 0.00 | 0.05 - 0.08 | 0.21 ± 0.34 | 0.00 - 1.58 |
|  | 11 | NG |  | NG |  | NG |  |
|  | 11.5 | NG |  | NG |  | NG |  |
|  | 12 | NG |  | NG |  | NG |  |
| M53 | 2 | NG |  | NG |  | NG |  |
|  | 2.5 | 0.65 ± 0.04 | 0.64 - 0.66 | 0.02 ± 0.00 | 0.02 - 0.02 | 4.96 ± 0.88 | 3.08 - 6.85 |
|  | 3 | 1.37 ± 0.10 | 1.36 - 1.39 | 0.12 ± 0.01 | 0.10 - 0.13 | 2.03 ± 0.41 | 1.43 - 2.63 |
|  | 4.5 | 1.73 ± 0.06 | 1.71 - 1.74 | 0.19 ± 0.01 | 0.16 - 0.22 | 1.88 ± 0.19 | 1.21 - 2.55 |
|  | 5.5 | 1.68 ± 0.08 | 1.67 - 1.70 | 0.20 ± 0.01 | 0.17 - 0.23 | 1.68 ± 0.26 | 1.06 - 2.30 |
|  | 8.5 | 1.62 ± 0.11 | 1.60 - 1.64 | 0.19 ± 0.04 | 0.16 - 0.22 | 2.30 ± 0.77 | 1.51 - 3.09 |
|  | 11 | NG |  | NG |  | NG |  |
|  | 11.5 | NG |  | NG |  | NG |  |
|  | 12 | NG |  | NG |  | NG |  |
| M57 | 2 | NG |  | NG |  | NG |  |
|  | 2.5 | 0.65 ± 0.17 | 0.42 - 0.88 | 0.01 ± 0.00 | 0.01 - 0.01 | 5.46 ± 1.34 | 2.49 - 8.42 |
|  | 3 | 0.68 ± 0.01 | 0.66 - 0.69 | 0.07 ± 0.00 | 0.06 - 0.08 | 2.94 ± 0.24 | 2.17 - 3.71 |
|  | 4.5 | 0.79 ± 0.01 | 0.78 - 0.81 | 0.13 ± 0.04 | 0.10 - 0.15 | 2.96 ± 0.40 | 2.37 - 3.55 |
|  | 5.5 | 0.79 ± 0.01 | 0.78 - 0.80 | 0.11 ± 0.01 | 0.10 - 0.12 | 2.17 ± 0.59 | 1.73 - 2.60 |
|  | 8.5 | 0.77 ± 0.05 | 0.75 - 0.78 | 0.12 ± 0.00 | 0.09 - 0.14 | 3.43 ± 0.08 | 2.72 - 4.14 |
|  | 11 | NG |  | NG |  | NG |  |
|  | 11.5 | NG |  | NG |  | NG |  |
|  | 12 | NG |  | NG |  | NG |  |
| SB31 | 2 | NG |  | NG |  | NG |  |
|  | 2.5 | 0.96 ± 0.02 | 0.95 - 0.97 | 0.02 ± 0.00 | 0.02 - 0.02 | 1.04 ± 1.51 | 0.00 - 2.46 |
|  | 3 | 1.39 ± 0.10 | 1.37 - 1.40 | 0.10 ± 0.00 | 0.09 - 0.11 | 2.03 ± 0.34 | 1.21 - 2.84 |
|  | 4.5 | 1.60 ± 0.01 | 1.59 - 1.62 | 0.19 ± 0.01 | 0.17 - 0.21 | 1.35 ± 0.16 | 0.81 - 1.89 |
|  | 5.5 | 1.64 ± 0.09 | 1.62 - 1.65 | 0.24 ± 0.03 | 0.20 - 0.28 | 1.83 ± 0.53 | 1.28 - 2.38 |
|  | 8.5 | 1.81 ± 0.06 | 1.78 - 1.83 | 0.16 ± 0.02 | 0.14 - 0.18 | 1.38 ± 0.36 | 0.53 - 2.23 |
|  | 11 | NG |  | NG |  | NG |  |
|  | 11.5 | NG |  | NG |  | NG |  |
|  | 12 | NG |  | NG |  | NG |  |
| SR8 | 2 | NG |  | NG |  | NG |  |
|  | 2.5 | NG |  | NG |  | NG |  |
|  | 3 | 1.73 ± 0.07 | 1.70 - 1.75 | 0.14 ± 0.02 | 0.12 - 0.17 | 2.98 ± 1.03 | 1.93 - 4.02 |
|  | 4.5 | 1.38 ± 0.05 | 1.37 - 1.39 | 0.21 ± 0.03 | 0.18 - 0.23 | 2.17 ± 0.52 | 1.70 - 2.63 |
|  | 5.5 | 1.47 ± 0.02 | 1.45 - 1.48 | 0.24 ± 0.02 | 0.20 - 0.28 | 2.29 ± 0.34 | 1.73 - 2.85 |
|  | 8.5 | 1.61 ± 0.07 | 1.60 - 1.62 | 0.23 ± 0.02 | 0.20 - 0.26 | 3.15 ± 0.11 | 2.65 - 3.65 |
|  | 11 | NG |  | NG |  | NG |  |
|  | 11.5 | NG |  | NG |  | NG |  |
|  | 12 | NG |  | NG |  | NG |  |
| SR350 | 2 | NG |  | NG |  | NG |  |
|  | 2.5 | 0.72 ± 0.08 | 0.71 - 0.72 | 0.03 ± 0.00 | 0.02 - 0.03 | 4.54 ± 0.16 | 3.71 - 5.37 |
|  | 3 | 1.63 ± 0.02 | 1.63 - 1.64 | 0.16 ± 0.01 | 0.16 - 0.17 | 3.76 ± 0.12 | 3.46 - 4.07 |
|  | 4.5 | 1.51 ± 0.07 | 1.50 - 1.53 | 0.17 ± 0.03 | 0.15 - 0.19 | 2.94 ± 0.10 | 2.38 - 3.51 |
|  | 5.5 | 1.56 ± 0.04 | 1.55 - 1.57 | 0.21 ± 0.02 | 0.19 - 0.23 | 2.86 ± 0.38 | 2.44 - 3.27 |
|  | 8.5 | 1.46 ± 0.09 | 1.45 - 1.47 | 0.24 ± 0.00 | 0.22 - 0.27 | 3.70 ± 0.44 | 3.35 - 4.05 |
|  | 11 | NG |  | NG |  | NG |  |
|  | 11.5 | NG |  | NG |  | NG |  |
|  | 12 | NG |  | NG |  | NG |  |
| T415 | 2 | NG |  | NG |  | NG |  |
|  | 2.5 | 0.83 ± 0.20 | 0.82 - 0.85 | 0.02 ± 0.01 | 0.02 - 0.02 | 2.04 ± 1.35 | 0.38 - 3.70 |
|  | 3 | 1.36 ± 0.05 | 1.35 - 1.38 | 0.12 ± 0.00 | 0.10 - 0.13 | 1.58 ± 0.22 | 0.83 - 2.33 |
|  | 4.5 | 1.60 ± 0.01 | 1.58 - 1.62 | 0.17 ± 0.02 | 0.15 - 0.20 | 1.87 ± 0.54 | 1.12 - 2.63 |
|  | 5.5 | 1.64 ± 0.07 | 1.62 - 1.66 | 0.19 ± 0.01 | 0.16 - 0.22 | 1.50 ± 0.31 | 0.87 - 2.12 |
|  | 8.5 | 1.53 ± 0.03 | 1.52 - 1.55 | 0.17 ± 0.01 | 0.15 - 0.19 | 2.11 ± 0.40 | 1.46 - 2.76 |
|  | 11 | NG |  | NG |  | NG |  |
|  | 11.5 | NG |  | NG |  | NG |  |
|  | 12 | NG |  | NG |  | NG |  |

Parameters: D is the maximum OD reached; µ_max_ denotes the maximum specific growth rate (h^-1^); and λ the lag phase period (h). Values represent the mean ± standard deviation from three independent replicates. NG: no growth.
